# Chemical Composition and Biological Properties of Two *Jatropha* Species: Different Parts and Different Extraction Methods

**DOI:** 10.3390/antiox10050792

**Published:** 2021-05-17

**Authors:** Gokhan Zengin, Mohamad Fawzi Mahomoodally, Kouadio Ibrahime Sinan, Gunes Ak, Ouattara Katinan Etienne, Jugreet B. Sharmeen, Luigi Brunetti, Sheila Leone, Simonetta Cristina Di Simone, Lucia Recinella, Annalisa Chiavaroli, Luigi Menghini, Giustino Orlando, József Jekő, Zoltán Cziáky, Claudio Ferrante

**Affiliations:** 1 Physiology and Biochemistry Research Laboratory, Department of Biology, Science Faculty, Selcuk University Campus, 42130 Konya, Turkey; sinankouadio@gmail.com (K.I.S.); akguneselcuk@gmail.com (G.A.); 2 Department of Health Sciences, Faculty of Medicine and Health Sciences, University of Mauritius, Réduit 230, Mauritius; f.mahomoodally@uom.ac.mu (M.F.M.); sharmeenjugs@gmail.com (J.B.S.); 3 Laboratoire de Botanique, UFR Biosciences, Université Félix Houphouët-Boigny, Abidjan 00225, Côte d’Ivoire; katinan.etienne@gmail.com; 4 Department of Pharmacy, Medicinal Plant Unit (MPU), Botanic Garden “Giardino dei Semplici”, “G. d’Annunzio” University of Chieti-Pescara, Via dei Vestini, 66100 Chieti, Italy; luigi.brunetti@unich.it (L.B.); sheila.leone@unich.it (S.L.); disimonesimonetta@gmail.com (S.C.D.S.); lucia.recinella@unich.it (L.R.); annalisa.chiavaroli@unich.it (A.C.); luigi.menghini@unich.it (L.M.); claudio.ferrante@unich.it (C.F.); 5 Agricultural and Molecular Research and Service Institute, University of Nyíregyháza, 4400 Nyíregyháza, Hungary; jjozsi@gmail.com (J.J.); cziaky.zoltan@nye.hu (Z.C.)

**Keywords:** *Jatropha* species, HPLC-MS/MS, phytochemicals, antioxidant, enzyme inhibitors, bioinformatics, gene expression

## Abstract

*Jatropha* L. species, in particular, *J. curcas* and *J. gossypiifolia*, are well known medicinal plants used for treating various diseases. In the present study, leaf and stem bark extracts of *J. curcas* and *J. gossypiifolia* obtained by maceration or homogenizer assisted extraction, were investigated for their phytochemical contents and biological potential as antioxidants, enzyme inhibitors and neuromodulators. In this regard, the gene expression of tumor necrosis factor α (TNFα) and brain-derived neurotrophic factor (BDNF) was investigated in hypothalamic HypoE22 cells. Finally, a bioinformatics analysis was carried out with the aim to unravel the putative mechanisms consistent with both metabolomic fingerprints and pharmacological effects. The leaf extracts of *J. curcas* showed higher total phenolic content (TPC) and total flavonoid content (TFC) than the stem bark extracts (range: 5.79–48.95 mg GAE/g and 1.64–13.99 mg RE/g, respectively), while *J. gossypiifolia* possessed TPC and TFC in the range of 42.62–62.83 mg GAE/g and 6.97–17.63 mg RE/g, respectively. HPLC-MS/MS analysis revealed that the leaf extracts of both species obtained by homogenizer assisted extraction are richer in phytochemical compounds compared to the stem bark extracts obtained by the same extraction method. In vitro antioxidant potentials were also demonstrated in different assays (DPPH: 6.89–193.93 mg TE/g, ABTS: 20.20–255.39 mg TE/g, CUPRAC: 21.07–333.30 mg TE/g, FRAP: 14.02–168.93 mg TE/g, metal chelating activity: 3.21–17.51 mg EDTAE/g and phosphomolybdenum assay: 1.76–3.55 mmol TE/g). In particular, the leaf extract of *J. curcas* and the stem bark extract of *J. gossypiifolia*, both obtained by homogenizer assisted extraction, showed the most potent antioxidant capacity in terms of free radical scavenging and reducing activity, which could be related to their higher TPC and TFC. Furthermore, anti-neurodegenerative (acetylcholinesterase inhibition: 1.12–2.36 mg GALAE/g; butyrylcholinetserase inhibition: 0.50–3.68 mg GALAE/g), anti-hyperpigmentation (tyrosinase inhibition: 38.14–57.59 mg KAE/g) and antidiabetic (amylase inhibition: 0.28–0.62 mmol ACAE/g; glucosidase inhibition: 0.65–0.81 mmol ACAE/g) properties were displayed differentially by the different extracts. Additionally, the extracts were effective in reducing the gene expression of both TNFα and BDNF, which could be partially mediated by phenolic compounds such as naringenin, apigenin and quercetin. Indeed, the scientific data obtained from the present study complement the several other reports highlighting the pharmacological potentials of these two species, thus supporting their uses as therapeutically active plants.

## 1. Introduction

The genus *Jatropha* L., which belongs to the tribe Joannesieae in the Euphorbiaceae family, contains approximately 170 known species. The name *Jatropha* is derived from the Greek word ‘‘jatros’’ (doctor) and ‘‘trophe”(food), which implies its medicinal uses [1]. *Jatropha* species are widely used in traditional folklore medicine to cure various ailments in Africa, Asia and Latin America and are also used as ornamental plants and energy crops [2]. 

*Jatropha* species have been used as medicinal plants by native people in many tropical and subtropical countries. For instance, *Jatropha* species are famous for the purgative effect of the seed oil. This purgative effect has been directed to cure digestive system symptoms like diarrhoea, dysentery, vomiting, retching and stomachache. Additionally, some parts of *Jatropha* plants are employed to heal skin-related ailments. The seed oil, leaf, latex, stem bark or root of *Jatropha* plants are pounded and applied on infected skin such as eczema, itches, mouth blisters, carbuncles, wounds and swellings. They are also believed to cure venereal diseases and urinary discharge. Moreover, the roots of some *Jatropha* species have long been applied on people suffering from leprosy and gonorrhea [3].

Several reviews have been conducted on the different species of the genus *Jatropha* covering various aspects such as their ethnobotany, medicinal properties, phytochemistry, and toxicity among others [3,4,5]. Phytochemical studies of the genus *Jatropha* have increased in recent years due to the high potential of these species as natural sources of bioactive compounds. Investigations of the chemical constituents of *Jatropha* plants resulted in the isolation of a number of alkaloids, cyclic peptides, terpenes (monoterpene, sesquiterpenes, diterpenes and triterpenes), flavonoids, lignans, coumarins, coumarino-lignoids, a non-cyanogenic glucoside, phloroglucinols, ester ferulates, phenolics, deoxypreussomerins and fatty acids [3]. Moreover, extracts and isolated compounds from various species of this genus have been found to possess properties of cytotoxicity, antimicrobial, anti-inflammatory, antioxidant, insecticidal, larvicidal, cholinesterase inhibition, and toxicity activities [6].

In particular, among the various *Jatropha* species, *J. gossypiifolia* has been documented to exhibit promising biological effects. For instance, its stem latex has been reported to possess coagulating features by reducing clotting and bleeding times in experiments, thereby providing a scientific basis for its use as a haemostatic agent [7]. Furthermore, jatrophone, an active compound isolated from *J. gossypiifolia*, has been reported to show a better anticancer effect against hepatocellular carcinoma (Hep G2 1886) compared to standard anticancer drugs like sorafenib and arsenic trioxyde [8].

Another important species of the genus *J. curcas* has also been appraised for its broad spectrum of pharmacological activities. As example, extracts of this plant were found to display antiviral activity on human immunodeficiency virus [9], while others reported remarkable anti-inflammatory and antibacterial, cosmetic and wound healing properties [10,11,12].

Therefore, taking into consideration the striking scientific data gathered so far, the present study was conducted to investigate the pharmacological properties further, in terms of the antioxidant, antidiabetic, anti-neurodegenerative and anti-hyperpigmentation, of methanolic extracts of different parts (leaf and stem bark) of *J. curcas* L. and *J. gossypiifolia* L., two important species of the genus *Jatropha* using different extraction methods (maceration and homogenizer assisted extraction). The protective and neuromodulatory effects of the extracts were evaluated in hypothalamic HypoE22 cells. In this regard, the gene expression of tumor necrosis factor α (TNFα) and brain-derived neurotrophic factor (BDNF) was measured. This study also attempted to analyze the total phenolic and flavonoid contents using spectrophometric analysis, as well as detect and characterize the phytochemical profiles of the extracts using HPLC-MS/MS. Finally, a bioinformatics analysis was carried out with the aim to unravel the putative mechanisms consistent with both metabolomic fingerprints and pharmacological effects. 

## 2. Materials and Methods

### 2.1. Plant Materials

The *Jatropha* species (*J. curcas* and *J. gossipiifolia*) were collected in the village of Lolodo (district of Yamoussoukro) of Côte d’Ivoire in the year 2019 and authenticated by the botanist Ouattara Katinan Etienne (Université Félix Houphouet Boigny, Abidjan, Ivory Coast). Voucher specimens were deposited in Science Faculty, Selcuk University. The stem bark and leaf samples were randomly collected from ten plants in the same population. The stem bark samples were stripped vertically while using a knife to limit it to the cambium layer. The plant materials were dried under shade for 10 days. 

### 2.2. Extraction

The plant materials were ground and then 10 g were extracted with methanol by using maceration (MAC) and homogenizer-assisted extraction (HAE) techniques. In MAC, the plant materials (5 g) were macerated with 100 mL of methanol at room temperature (about 25 ± 2 °C) for 24 h. Regarding HAE, the plant materials (5 g) were extracted with methanol (100 mL) by using one ultra-turrax (6000× *g*) for 5 min at room temperature (about 25 ± 2 °C). All extracts were filtered by using Whatman No.1 filter papers and then the extracts were evaporated to dryness and stored at 4 °C until analysis. 

### 2.3. Total Phenolic and Flavonoid Content

Spectrophotometric methods were used to determine total phenolic and flavonoid content as conducted previously. Standard equivalents (gallic acid equivalent (GAE) for phenolic and rutin equivalent (RE) for flavonoid) were used to assess the bioactive content in the plant extracts [13,14].

### 2.4. HPLC Analysis

Chromatographic separation was accomplished with a Dionex Ultimate 3000RS HPLC instrument, equipped with a Thermo Accucore C18 (100 mm × 2.1 mm i. d., 2.6 μm) analytical column for separation of compounds. Water (A) and methanol (B) containing 0.1% formic acid were employed as mobile phases, respectively. The total run time was 70 min; the elution profile and all exact analytical conditions have been published [15].

### 2.5. Determination of Antioxidant and Enzyme Inhibitory Effects

Antioxidant protocols included reducing power (cupric reducing antioxidant capacity (CUPRAC) and ferric reducing power (FRAP)), metal chelating, phosphomolybdenum (PBD) and free radical scavenging (2,2-diphenyl-1-picrylhydrazyl (DPPH) and 3-ethylbenzothiazoline-6-sulphonic acid (ABTS)) activities. Experimental details were as described previously by [16]. Inhibitory effects of the extracts were tested against different enzymes (tyrosinase, α-amylase, α-glucosidase and cholinesterases). Trolox and ethylenediaminetetraacetic acid (EDTA) for antioxidant, galantamine for cholinesterases, kojic acid for tyrosinase, and acarbose for α-amylase and α-glucosidase were used to express antioxidant and enzyme inhibitory results.

In the antioxidant and enzyme inhibitory assays, one-way ANOVA with Tukey comparison test were performed to display significance level among the extracts at a confidence level of 95%. Xlstat 2016 was used for statistical analyses. 

### 2.6. Artemia salina Lethality Bioassay

*Artemia salina* cysts were hatched in oxygenated artificial sea water (1 g cysts/L). After 24 h, brine shrimp larvae were gently transferred with a pipette into 6-well plates containing 2 mL of herbal extracts at different concentrations (0.1–20 mg/mL) in artificial sea water. Ten larvae per well were incubated at 25–28 °C for 24 h. After 24 h, the number of living napulii were counted under light microscope and compared to the control untreated group. Results were expressed as percentage of mortality calculated as: ((T − S)/T) ∗ 100. T is the total number of incubated larvae and S is the number of survival napulii. Living nauplii were considered those exhibiting light activating movements during 10 s of observation. For each experimental condition, two replicates per plate were performed and experimental triplicates were performed in separate plates.

### 2.7. Cell Cultures and Viability Test

HypoE22 cells were purchased from Cedarlane Cellution Biosystem and cultured in DMEM (Euroclone) supplemented with 10% (*v*/*v*) heat-inactivated fetal bovine serum and 1.2% (*v*/*v*) penicillin G/streptomycin in 75 cm^2^ tissue culture flasks (*n* = 5 individual culture flasks for each condition). The cultured cells were maintained in a humidified incubator with 5% CO^2^ at 37 °C. For cell differentiation, cell suspension at a density of 1 × 10^6^ cells/mL was treated with various concentrations (10, 50, and 100 ng/mL) of phorbol myristate acetate (PMA, Fluka) for 24 h or 48 h (induction phase). Thereafter, the PMA-treated cells were washed twice with pH 7.4 phosphate buffer solution (PBS) to remove PMA and non-adherent cells, whereas the adherent cells were further maintained for 48 h (recovery phase). Morphology of cells was examined under an inverted phase-contrast microscope. To assess the basal cytotoxicity of herbal extract, a viability test was performed on 96 microwell plates, using the 3-(4,5-dimethylthiazol-2-yl)-2,5-diphenyltetrazolium bromide (MTT) test. Cells were incubated with extracts (ranging concentration 1–100 µg/mL) for 24 h. After the treatment period, 10 μL of MTT (5 mg/mL) were added to each well and incubated for 3 h. The formazan dye formed was extracted with dimethyl sulfoxide and absorbance recorded. Effects on cell viability were evaluated in comparison to the untreated control group. 

### 2.8. RNA Extraction, Reverse Transcription, and Real-Time Reverse Transcription Polymerase Chain Reaction (Real-Time RT PCR)

Total RNA was extracted from the cells using TRI Reagent (Sigma-Aldrich, St. Louis, MO, USA), according to the manufacturer’s protocol. Contaminating DNA was removed using two units of RNase-free DNase 1 (DNA-free kit, Ambion, Austin, TX, USA). The RNA solution was quantified at 260 nm by spectrophotometer reading (BioPhotometer, Eppendorf, Hamburg, Germany) and its purity was assessed by the ratio at 260 and 280 nm readings. The quality of the extracted RNA samples was also determined by electrophoresis through agarose gels and staining with ethidium bromide under UV light. Ine microgram of total RNA was reverse transcribed using a High Capacity cDNA Reverse Transcription Kit (Applied Biosystems, Foster City, CA, USA). Reactions were incubated in a 2720 Thermal Cycler (Applied Biosystems, Foster City, CA, USA) initially at 25 °C for 10 min, then at 37 °C for 120 min, and finally at 85 °C for 5 s. Gene expression was determined by quantitative real-time PCR using TaqMan probe-based chemistry (Applied Biosystems, Foster City, CA, USA). PCR primers and TaqMan probes were obtained from Applied Biosystems (Assays-on-Demand Gene Expression Products, Rn02531967_s1 for BDNF gene; Rn01525859_g1 for TNF-α). β-actin (Applied Biosystems, Foster City, CA, USA, Part No. 4352340E) was used as the housekeeping gene. The real-time PCR was carried out in triplicate for each cDNA sample in relation to each of the investigated genes. Data were elaborated with the Sequence Detection System (SDS) software version 2.3 (Applied Biosystems, Foster City, CA, USA).

### 2.9. Bioinformatics

The chemical structures were prepared with ChemSketch software and the related canonical SMILES were then processed by the STITCH platform, for predicting putative pharmacological targets. The identification of predicted targets was confirmed through the use of UniProt database. Protein–protein interactions were predicted through STRINGH bioinformatics platform. Docking calculations were conducted through the Autodock Vina of PyRx 0.8 software. Crystal structures of target proteins were derived from the Protein Data Bank (PDB) with PDB IDs as follows: 5FDR (Induced myeloid leukemia cell differentiation protein (MCL1)); 1QKU (Estrogen receptor 1 (ESR1)). Discovery studio 2020 visualizer was employed to investigate the protein–ligand non-bonding interactions.

### 2.10. Statistical Analysis

GraphPad Prism for Windows v5.01 (GraphPad Software, San Diego, CA, USA) was used to analyze the experimental results. The means ± SD were determined for each experimental group and analyzed using one-way analysis of variance (ANOVA), followed by a Newman–Keuls comparison multiple test.

## 3. Results and Discussion

In the present study, two extraction methods, namely maceration and homogenizer assisted extraction were used to see if there was an effect on the yield of bioactive compounds and biological properties of the extracts. The maceration technique was selected to preserve thermolabile compounds in the tested plant materials. Regarding homogenizer assisted extraction, this technique was used as one of green extraction techniques with shorter extraction time. Thus, the traditional (maceration) and green extraction (homogenizer assisted extracts) methods were compared. 

Spectrophotometry is one of the relatively simple techniques for quantification of plant total phenolics and total flavonoids [17]. In the present study, spectrophotometric determination of extracts of *J. curcas* were found to possess significantly higher total phenolic contents (TPC) in the leaf extracts than stem bark extracts (range: 5.79–48.95 mg GAE/g). Conversely, the highest TPC was yielded in the stem bark extract of *J. gossypiifolia* obtained by homogenizer assisted extraction (62.83 ± 2.05 mg GAE/g) compared with the other extracts of the plant (42.62–49.05 mg GAE/g) (Table 1). 

A similar trend was noted for the extracts with regard to their contents of total flavonoids. For instance, the leaf extracts of *J. curcas* showed significantly higher total flavonoid contents (TFC) than the stem bark extracts (range: 1.64–13.99 mg RE/g). On the other hand, for *J. gossypiifolia*, the highest and lowest TFC were yielded by the stem bark extract and leaf extract, respectively, both obtained by homogenizer assisted extraction (17.63 ±0.34 mg RE/g and 6.97 ± 0.32 mg RE/g, respectively). The leaf and stem bark extracts of *J. gossypiifolia* obtained by maceration showed TFC 11.04 ± 0.59 mg RE/g, and 12.71 ± 0.10 mg RE/g, respectively (Table 1).

In particular, *J. gossypiifolia* was found to yield the highest TPC and TFC when homogenizer assisted extraction was used. Indeed, other studies have also shown homogenizer assisted extraction to present high potential for extracting phenolics and antioxidant compounds [18]. Interestingly, several studies have also demonstrated that extraction techniques play a crucial role in the yield of phenolic content from plant extracts [19,20].

Other researchers also determined the TPC and TFC from different parts of *J. curcas* and *J. gossypiifolia*. For instance, investigation of the methanolic extracts of *J. gossypiifolia* revealed the leaves to have higher total phenolic content (65.66 mg GAE/g) compared to the stem portion (33.332 mg GAE/g) [21]. Additionally, the total phenolic content of crude extract *J. curcas* fruit was found to possess TPC 7.04 ± 0.10 mg GAE/g of extract and 0.22–18.61 mg GAE/g of extract for its fraction [22]. The polyphenolic contents of the ethanol, methanol and aqueous extracts of the stem bark of *J. curcas* were also assessed by Igbinosa, et al. [23], whereby the total phenol and total flavonoid were obtained in amounts of 10.92–28.87 mg tannic acid/g extract and 6.28–11.18 mg quercetin/g extract, respectively.

HPLC-MS/MS analysis was also performed on extracts obtained by homogenizer assisted extraction. A total of 68 compounds were revealed to be present in the leaf extract of *J. curcas*, whereas only 44 compounds were detected in the stem bark extract. However, many compounds were found in both extracts, such as loliolide, orientin, soorientin, vitexin, isovitexin, isoquercitrin, quercetin, jasmonic acid, luteolin, sebacic acid, apigenin, 12-oxo phytodienoic acid, hydroxyoctadecatrienoic acid, hydroxyoctadecadienoic acid, hydroxyhexadecenoic acid, α-linolenic acid, linoleic acid, palmitic acid, oleic acid and stearic acid (Table 2 and Table 3). Detailed chemical composition is also available as Supplementary Materials.

On the other hand, 78 compounds were identified in *J. gossypifolia* leaf extract obtained by homogenizer assisted extraction, while 64 compounds were detected in the stem bark extract of *J. gossypifolia* obtained by the same method. Many compounds were also found to be present in both extracts of *J. gossypiifolia*, such as quinic acid, catechin, epiatechin, scopoletin, ferulic acid, loliolide, vicenin-1, orientin, vicenin-3, vitexin, isoorientin, dihydrokaempferol, isovitexin, luteolin-7-O-glucoside, isoquercitrin, quercetin, dodecanedioic acid, undecanedioic acid, isorhamnetin, apigenin, sebacic acid, naringenin, jasmonic acid, luteolin, kaempferol, hydroxyoctadecatrienoic acid, hydroxyoctadecadienoic acid, α-linolenic acid, linoleic acid, palmitic acid, oleic acid, stearic acid, 12-oxo phytodienoic acid, stearidonic acid, and 12-oxo phytodienoic acid (Table 4 and Table 5).

Indeed, for both studied *Jatropha* species, HPLC-MS/MS analysis showed the leaf extracts to be richer in phytochemical compounds compared to the stem bark extracts. However, the chemical profiles of both *Jatropha* species indicate that some compounds were uniformly distributed throughout the plant, that is the leaves and the stem bark. It has been suggested that some compounds are more concentrated in the roots and seeds and others in the green tissues of the aerial part such as stems and leaves. This is because each organ has a specialization that it must fulfill according to its physiological function. Interestingly however, in a previous study, the contents of each phenolic compound from the leaves and stems of two other *Jatropha* species, *J. cinerea* and *J. cordata* were found to significantly differ between species and plant organs [28]. Similarly, aqueous leaf extracts of *J. gossypiifolia* and *J. mollissima* prepared by decoction showed quantitatively different chemical profiles by HPLC-DAD [29].

Antioxidant properties of the tested extracts were investigated by different methods and the results are summarized in Table 5. In the present study, all extracts were found to possess free radical scavenging ability in both DPPH and ABTS assays. In the case of *J. curcas* extracts, the scavenging capacity in the DPPH assay ranged from 6.89 to124.70 mg TE/g, whereas in the ABTS assay, it ranged from 20.20 to 149.12 mg TE/g. For *J. gossypiifolia* extracts, the scavenging potential ranges were 48.14–193.93 mg TE/g and 86.88–160.00 mg TE/g in DPPH and ABTS assays, respectively. Interestingly, the leaf extracts of *J. curcas* were observed to exhibit significantly higher scavenging activity than the stem bark extracts, with the leaf extract obtained by the HAE method showing the highest activity. On the other hand, the stem bark-HAE extract of *J. gossypiifolia* was found to be the most prominent radical scavenger (Table 5).

In the present work, the extracts of *J. curcas* showed reducing activity of 21.07–256.21 mg TE/g and 14.02–97.03 mg TE/g in CUPRAC and FRAP assays, respectively. Remarkably, the same trend as in the radical scavenging assays (DPPH and ABTS) could be observed in the reducing assays (CUPRAC and FRAP). The leaf extracts of *J. curcas* showed better reducing activity compared to the stem bark extracts in both CUPRAC and FRAP assays. As for *J. gossypiifolia* extracts, reducing activities of 243.59–333.30 mg TE/g and 101.32–168.93 mg TE/g were obtained in CUPRAC and FRAP assays, respectively, with the highest activity displayed by stem bark-HAE extract (Table 6).

Moreover, the extracts of both species were found to act as metal chelators (*J. curcas*: 3.21–10.98 mg EDTAE/g; *J. gossypiifolia*: 13.67–18.98 mg EDTAE/g). However, it was revealed that the leaf extracts of both *J. curcas* and *J. gossypiifolia* showed higher metal chelating activity compared to the stem bark extracts (Table 6). Interestingly, this could be due to the higher number of phytochemicals detected in the leaf extracts obtained by the homogenizer assisted extraction compared to the stem bark extracts.

In the phosphomolybdenum assay, the highest total antioxidant capacity was shown by stem bark extracts of *J. curcas* (3.55 mM TE/g and 3.34 mM TE/g in extracts obtained by maceration and homogenizer assisted extraction, respectively), in contrast to the leaf extracts of *J. curcas* (2.27 and 2.57 mM TE/g). The total antioxidant capacity of the *J. gossypifolia* extracts ranged from 1.76 to 2.44 mM TE/g, with the lowest and highest activity demonstrated by stem bark/maceration and leaf/homogenizer assisted extraction extracts respectively (Table 6).

Numerous previous studies have also confirmed the antioxidant potential of *J. curcas* and *J. gossypiifolia* using various experimental models. For instance, using DPPH assay, Rofida [30] determined the antioxidant activity of ethanolic leaf, fruit, stem bark and root extracts of *J. curcas* (IC_50_: 26.44–420.98 µg/mL) and *J. gossypiifolia* (IC_50_: 10.79–98.63 µg/mL), obtained by maceration. Furthermore, the results showed that *J. curcas* stem bark extract possessed higher antioxidant activity, whereas in *J. gossypiifolia*, the leaves and stem bark extracts displayed better antioxidant activity [30]. In addition, based on phosphomolybdate assay and DPPH radical scavenging activity, the ethyl acetate extract of *J. gossipiifolia* was found to have high antioxidant activity when compared to other extracts studied by Saishri, et al. [31]. Even though the extract yield of ethyl acetate extract (4.6%) was lower when compared to the yield of ethanol extract (9.6%) and water extract (18%), the high antioxidant power exhibited by the ethyl acetate extract was suggested to be due to the presence of bioactive constituents.

Moreover, in the study of Saosoong, Litthanapongsatorn and Ruangviriyachai [22], the antioxidant activity of the crude extract of *J. curcas* fruit was found to be 270.98 ± 0.59 μmol Fe/g of extract using the phenanthroline method, while the extract gave an IC_50_ of 14.09 ± 0.05 mg/mL with the DPPH method. In particular, the methanolic fraction showed the highest antioxidant activity with an IC_50_ of 0.04 ± 0.02 mg/mL with the DPPH method and an antioxidant activity of 207.53 ± 2.58 μmol Fe/g of extract with the phenanthroline method. A good correlation among antioxidant activity in both methods and total phenolic content was also observed. 

In fact, significant strong correlations have been previously established between TPC and antioxidant potentials of plant extracts, signifying that the polyphenolic compounds present in the plant extracts contributed to their antioxidant activity and reducing capability [32]. These findings were in agreement with the results of the present study, showing extracts with higher TPC exhibiting higher antioxidant activity.

Cholinesterase inhibitors function by inhibiting cholinesterase from hydrolyzing acetylcholine into its components of acetate and choline. This allows for an increase in the availability and duration of action of acetylcholine in neuromuscular junctions. Most commonly, their use is in treating neurogenerative diseases such as Alzheimer disease, Parkinson disease, and Lewy body dementia. Indeed, plants have been widely assessed as potent sources of natural cholinesterase inhibitors [33,34]. In the present study, while the leaf extract of *J. curcas* obtained by maceration did not show any AChE inhibition, leaf extracts obtained by homogenizer assisted extraction and stem bark extracts of *J. curcas* showed AChE inhibitory potential ranging from 2.04 to 2.36 mg GALAE/g. Comparatively, all extracts of *J. gossypifolia* were found to be active as AChE inhibitors (1.12–2.06 mg GALAE/g). Additionally, BChE inhibition was exhibited by all extracts of *J. curcas*, with the stem bark extracts showing higher potential than leaf extracts (1.59–3.68 mg GALAE/g). However, with the exception of the leaf/maceration extract of *J. gossypifolia*, which showed no activity against BChE, all other extracts of *J. gossypifolia* were found to inhibit BChE with an inhibition range of 0.50–0.72 mg GALAE/g (Table 6).

Eighteen species belonging to *Convolvulaceae*, *Crassulaceae*, *Euphorbiaceae*, *Leguminosae, Malvaceae, Moraceae, Nyctaginaceae* and *Rutaceae* families were tested for their anti-AChE in the study of Feitosa, et al. [35], whereby among the most active plants, *J. curcas* (IC_50_ = 0.25 mg/mL) and *J. gossypiifolia* (IC_50_ = 0.05 mg/mL) were also found to possess promising anti-AChE activity compared to galantamine (IC_50_ = 0.37 × 10^−3^ mg/mL). The authors suggested that there could be compounds with a similar activity to galanthamine present in the plant extracts. Saleem, et al. [36] also reported the cholinesterase inhibitory potentials of *J. gossypiifolia*. For instance, the root dichloromethane fraction (% inhibition: 65.43 ± 0.11%), root methanol fraction (62.79 ± 0.34%) and leaf dichloromethane fraction (57.71 ± 0.15%) of *J. gossypiifolia* showed significant AChE inhibitory activity relative to other tested fractions when compared with the standard, eserine (91.29 ± 1.17%). Furthermore, BChE enzyme inhibitory results showed that the root dichloromethane fraction (80.46 ± 0.44%) and leaf ethyl acetate extract (77.34 ± 0.34%) displayed significant BChE enzyme inhibitory activity relative to other tested fractions when compared with the standard, eserine (82.82 ± 1.09%). 

Tyrosinase (EC 1.14.18.0) is a copper-containing mixed-function oxidase that is ubiquitously expressed in animals, plants, and microorganisms. Furthermore, tyrosinase is a key rate-limiting enzyme that can catalyze enzyme browning and melanin synthesis. In humans, the overexpression of tyrosinase leads to the overproduction of melanin in the skin, which can trigger hyperpigmentation effects such as melasma, freckles, age spots, and melanoma [37]. In the present study, all extracts of *J. curcas* and *J. gossypiifolia* displayed anti-tyrosinase potential (*J. curcas*: 38.14–56.30 mg KAE/g; *J. gossypiifolia*: 50.43–57.59 mg KAE/g). However, while the leaf extracts of *J. curcas* exhibited the most potent activity against tyrosinase, the highest anti-tyrosinase effect was shown by the stem bark extracts of *J. gossypiifolia* (Table 6).

Interestingly, the higher anti-tyrosinase effect shown by the *J. curcas* leaf extracts and *J. gossipiifolia* extracts in the present study, were found to be correlated with the high antioxidant potentials of those extracts. In fact, an extremely interesting and delicate relationship exists between antioxidant defense systems and melanogenesis. This relationship is associated with ROS scavenging. The synergistic effect in this relationship increases the effectiveness of antioxidants in scavenging free radicals, while tyrosinase inhibitors work, thus reducing melanin production [38]. Additionally, in a previous study, the fraction of water extracts of new and fallen *Sapium sebiferum* (L.) Roxb. leaves were found to possess great antioxidant and tyrosinase inhibition activities, even better than those of the positive control (BHT and arbutin). Moreover, the tyrosinase inhibition effect was significantly and positively correlated with its copper chelating activity, which was suggested to be the mechanism of tyrosinase inhibition [39].

There are numerous conventional drugs available for diabetes mellitus, which vary in their mechanism of action. One of the pharmacological approaches is by using carbohydrate enzyme inhibitor drugs such as acarbose, voglibose and miglitol. These drugs inhibit both α-amylase and α-glucosidase, which are enzymes responsible for the breakdown of carbohydrates. However, these current antidiabetic drugs suffer from a number of undesirable side effects, leading researchers to seek traditional medicinal plants as alternatives for diabetic treatment [32]. In the current work, all of the extracts of *J. curcas* acted as dual inhibitors of amylase (0.28–0.62 mmol ACAE/g) and glucosidase (0.63–0.81 mmol ACAE/g). While the leaf extracts of *J. curcas* showed greater inhibition against amylase than the stem bark extracts; however, the stem bark extracts of *J. curcas* were found to display a better glucosidase inhibitory effect compared to the leaf extracts. On the other hand, with the exception of the stem bark-HAE extract of *J. gossypifolia*, which selectively inhibited amylase (0.49 ± 0.01 mmol ACAE/g), all of the other extracts of *J. gossipifolia* showed dual inhibition against the carbohydrate hydrolyzing enzymes (0.43–0.81 mmol ACAE/g) (Table 6). 

Different extracts and fractions of the root, leaf and stem bark of *J. gossypiifola* were also screened for their α-glucosidase inhibitory property. n-Butanol and ethyl acetate fractions showed maximum enzyme inhibition for α-glucosidase with 67.93 ± 0.66 and 67.67 ± 0.71% and an IC_50_ of 218.47 ± 0.23 and 213.45 ± 0.12 μg/mL, respectively, while acarbose, used as a positive control, exhibited enzyme inhibition activity of 92.14 ± 0.38% with an IC_50_ of 38.24 ± 0.1 μg/mL [36]. 

**Figure 1:**
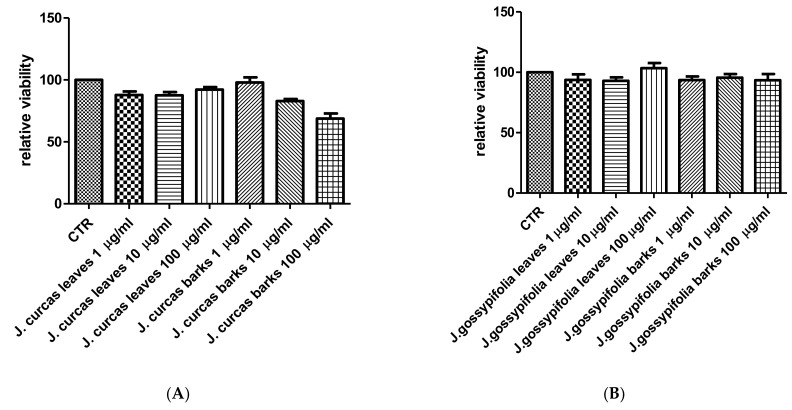
Null effect induced by the extracts (1–100 µg/mL) of *J. curcas* (**A**) and *J. gossypiifolia* (**B**) on HypoE22 cell viability.

**Figure 2:**
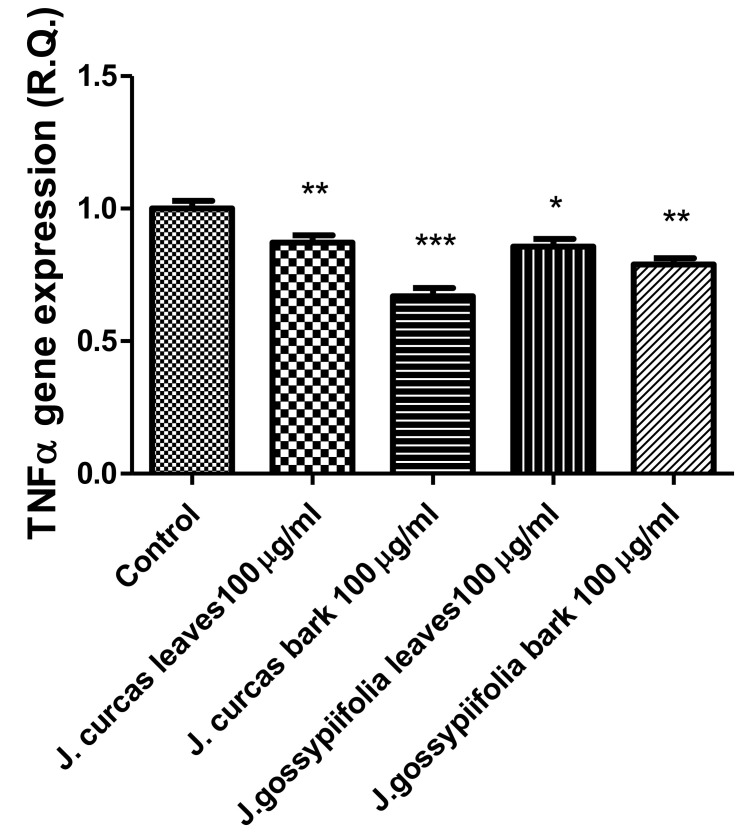
Inhibitory effects of *J. curcas* and *J. gossypiifolia* (100 µg/mL) on TNFα gene expression, in HypoE22 cells. ANOVA, *p* < 0.0001; *** *p* < 0.001, ** *p* < 0.01, * *p* < 0.05 vs. Control.

**Figure 3:**
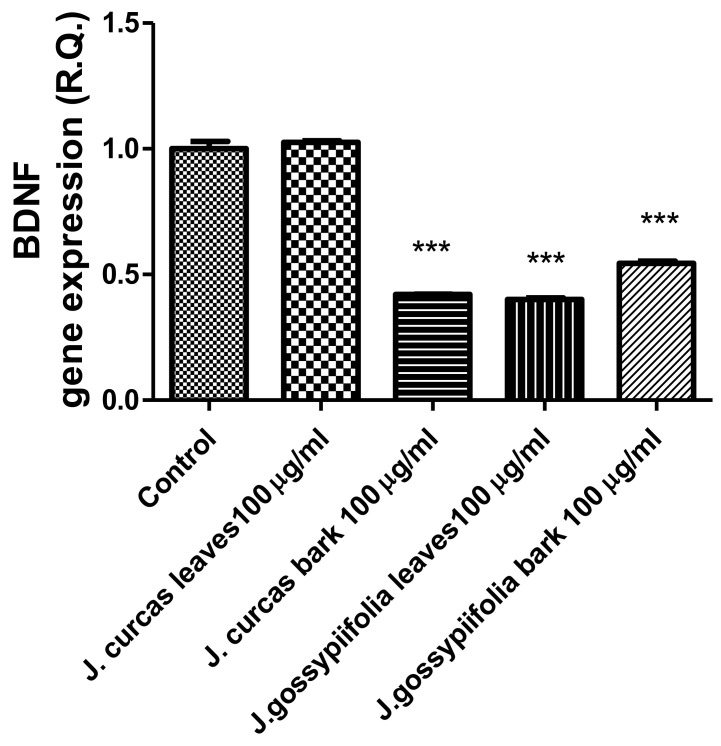
Inhibitory effects of *J. curcas* and *J. gossypiifolia* (100 µg/mL) on BDNF gene expression, in HypoE22 cells. ANOVA, *p* < 0.0001; *** *p* < 0.001 vs. Control.

**Figure 4:**
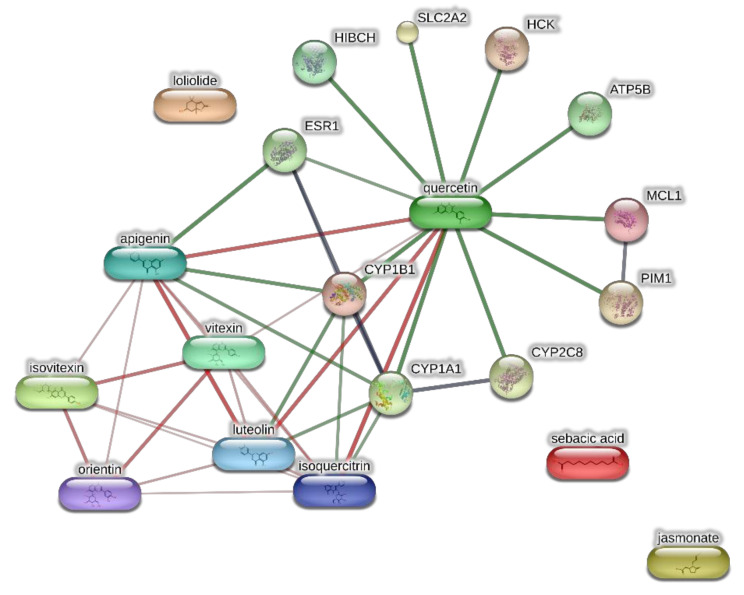
Components-targets analysis conducted through the bioinformatics platform STITCH for unravelling putative targets underlying the pharmacological effects on the extracts of *J. curcas.* The network pharmacology approach considered the most representative phytocompounds of the extracts, namely loliolide, orientin, soorientin, vitexin, isovitexin, isoquercitrin, quercetin, jasmonic acid, luteolin, sebacic acid, and apigenin.

**Figure 5:**
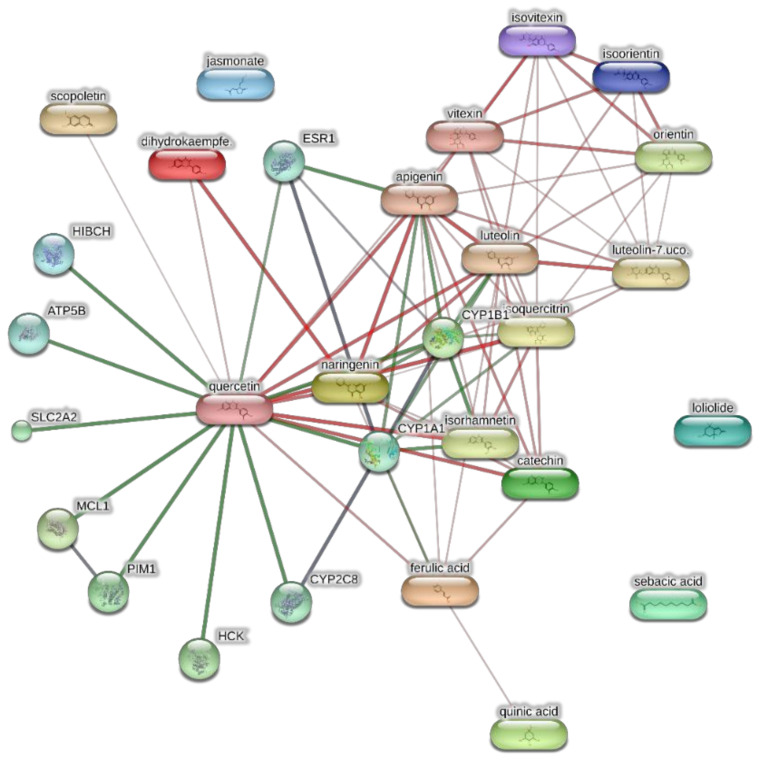
Components-targets analysis conducted through the bioinformatics platform STITCH for unravelling putative targets underlying the pharmacological effects on the extracts of *J. gossypiifolia.* The network pharmacology approach considered the most representative phytocompounds of the extracts, namely quinic acid, catechin, epiatechin, scopoletin, ferulic acid, loliolide, vicenin-1, orientin, vicenin-3, vitexin, isoorientin, dihydrokaempferol, isovitexin, luteolin-7-O-glucoside, isoquercitrin, quercetin, isorhamnetin, apigenin, sebacic acid, naringenin, jasmonic acid, and luteolin.

**Figure 6:**
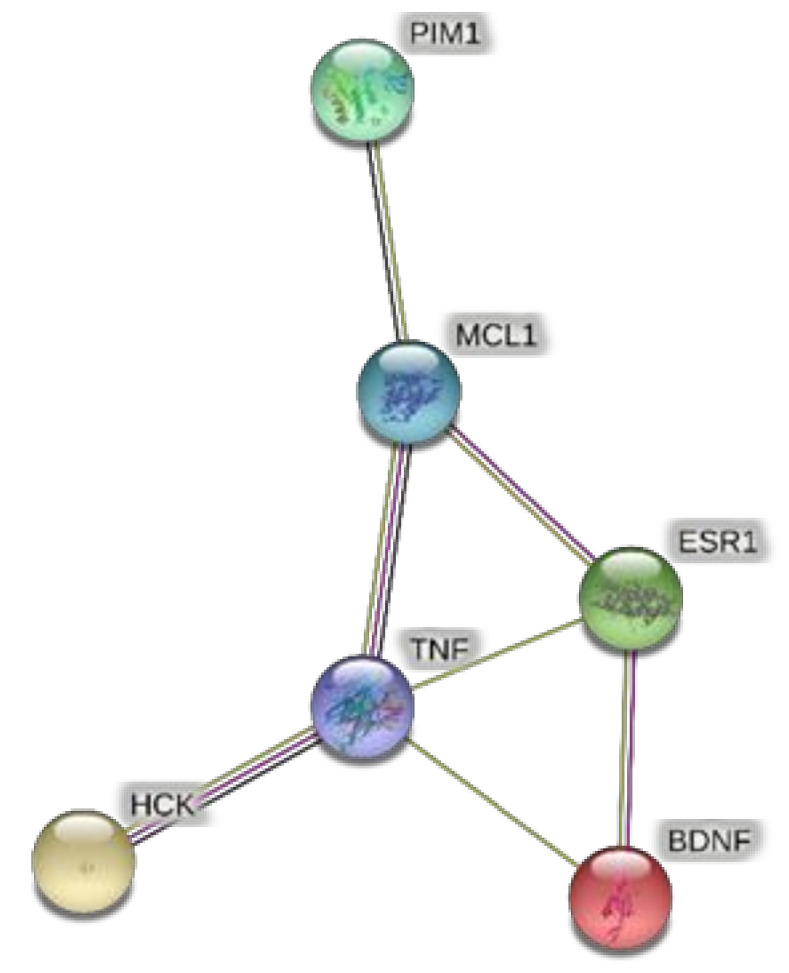
Protein–protein interactions predicted through the bioinformatics platform STRINGH. The bioinformatics resource showed interactions of BDNF with ESR1. While TNFα was predicted to interact with both HCK and MCL1. Considering the expression of ESR1 and MCL1 in the hypothalamus, the present bioinformatics prediction suggests that ESR1 and MCL1 could be targets underlying the modulation of BDNF and TNFα induced by the extracts, in HypoE22 cells.

**Figure 7:**
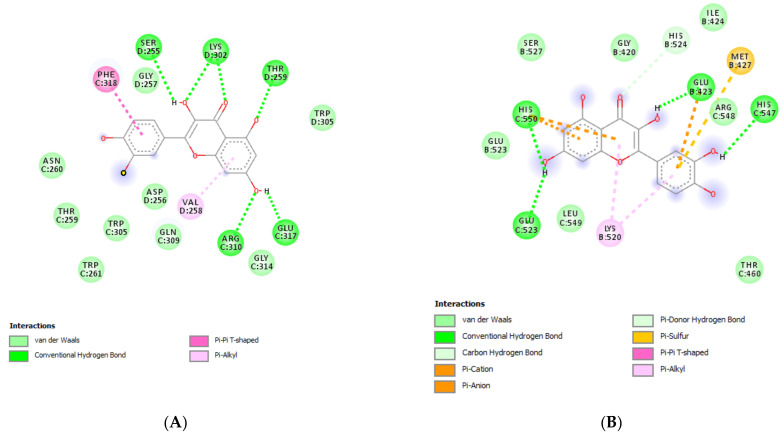
(**A**) Putative interactions between quercetin and myeloid cell leukemia 1 (MCL1; PDBID: 5FDR). Free energy of binding (ΔG) and affinity (Ki) are −7.8 kcal/mol and 1.9 µM, respectively. (**B**) Putative interactions between quercetin and estrogen receptor 1 (ESR1; PDBID: 1QKU). Free energy of binding (ΔG) and affinity (Ki) are −7.8 kcal/mol and 1.9 µM, respectively.

The extracts from *J. curcas* and *J. gossypiifolia* have been tested in the brine shrimp (*Artemia salina*) lethality test, which represents a valuable experimental model for predicting the limits of toxicity and biocompatibility in eukaryotic cells [40]. Specifically, the shrimp were exposed to scalar concentrations (0.1–20 mg/mL) of the extracts and the resulting LC_50_ values < 1 mg/mL indicate a high degree of toxicity in the nauplii. Although toxicological studies are still lacking for both *Jatropha* species, we cannot exclude that this intrinsic toxicity of the extracts could be related, at least in part, to the presence of terpenes, such as curcusones, but also flavonoids and saponins that could induce genotoxicity [41,42]. Considering the LC_50_ values yielded by the brine shrimp test, extract concentrations at least 10-fold lower (100 µg/mL) were chosen for the subsequent pharmacological tests [43]. Considering the intrinsic scavenging/reducing and anticholinesterase properties shown by the present extracts, the pharmacological assays were conducted using the non-tumoral hypothalamic HypoE22 cell line, which was demonstrated to be a useful experimental paradigm for investigating anti-inflammatory and neuromodulatory effects induced by herbal extracts [44]. Specifically, the HypoE22 cells were exposed to the extracts (1–100 µg/mL), and the cell viability was measured via MTT test, which showed a good tolerability of the hypothalamic cells at all tested concentrations. Indeed, the cell viability was always ≥70% (Figure 1A,B) compared to the untreated control group, and this was considered as an index of cell tolerability to the extract exposition in the 24 h following treatment [45]. Considering the results of the MTT test, the extract concentration of 100 µg/mL was chosen for the second set of experiments aiming to investigate the anti-inflammatory and neuromodulatory effects of the extracts. In this regard, the gene expression of TNFα and BDNF was measured, finding a significant reduction. Regarding the inhibition of TNFα (Figure 2), this is consistent, albeit partially, with the scavenging/reducing properties of the present extracts, but also with previous studies highlighting the capability of herbal extracts, with intrinsic antioxidant effects, to inhibit the gene expression of TNFα in HypoE22 cells [46]. However, the inhibition of the gene expression of BDNF (Figure 3), a neuropeptide playing a master role in neuroprotection [47], is discrepant with the effects of the extracts on TNFα and also with their antiradical properties. Nevertheless, we should consider that BDNF is also involved in the hypothalamic appetite-regulating network [48], with anorexigenic effects induced by its central administration [49]. The plasma levels of BDNF were lower in people suffering from anorexia, compared to healthy subjects, whereas the BDNF concentration tends to arise after normalization of body weight [50]. In this context, we hypothesize that BDNF modulation could be involved in the anorexigenic effect induced by *J. curcas* administration in rats [51]. Considering the results of the qualitative fingerprint analysis, a bioinformatics approach was conducted with the aim to identify the putative targets underlying the observed effects. In the case of *J. curcas*, the bioinformatics analysis, carried out on the platform STITCH, considered the following phytochemicals: loliolide, orientin, soorientin, vitexin, isovitexin, isoquercitrin, quercetin, jasmonic acid, luteolin, sebacic acid, and apigenin, present in the extracts from all *J. curcas* plant materials tested in the present study (Figure 4). While in the case of *J. gossypifolia*, the selected phytochemicals were quinic acid, catechin, epiatechin, scopoletin, ferulic acid, loliolide, vicenin-1, orientin, vicenin-3, vitexin, isoorientin, dihydrokaempferol, isovitexin, luteolin-7-O-glucoside, isoquercitrin, quercetin, isorhamnetin, apigenin, sebacic acid, naringenin, jasmonic acid, and luteolin (Figure 5). The bioinformatics predictions indicated, among the selected phytochemicals, prominent positions of quercetin, apigenin and naringenin in the scenario of putative interactions. Specifically, all of them were predicted to interact with estrogen receptor 1 (ESR1), whereas the sole apigenin displayed putative interactions with tyrosine-protein kinase HCK (HCK), playing a key role in regulating the innate immune response and with the apoptosis marker myeloid cell leukemia 1 (MCL1). Both ESR1 and MCL1 are expressed in the hypothalamus [52,53], whereas the bioinformatics platform STRINGH highlighted putative interactions with BDNF and TNFα (Figure 6). Therefore, the present bioinformatics analysis suggests that ESR1 and MCL1 could be targets of the selected phenolic compounds for mediating, at least in part, the inhibition of the gene expression of both BDNF and TNFα in the hypothalamus. In this regard, docking runs were also conducted to calculate the putative affinities of quercetin towards ESR1 and MCL1. The results of docking experiments (Figure 7A,B) showed identical micromolar affinities (1.9 µM) of quercetin towards the selected proteins. In the case of MCL1, the quercetin affinity is mainly due to the formation of hydrogen bonds with the protein, whereas pi-interactions also seem to be involved in the binding of quercetin with ESR1. Overall, these results further suggest that the present target proteins are crucial for mediating the observed pharmacological effects in the hypothalamus.

## 4. Conclusions

This study demonstrated the multifunctional potential of two *Jatropha* species, *J. curcas* and *J. gossypiifolia* as antioxidant, antidiabetic, anti-neurodegenerative and anti-hyperpigmenting agents. Moreover, the spectrophotometric coupled with HPLC-MS analysis revealed the plants to contain notable bioactive compounds that could have resulted in the biological properties demonstrated herein. This was most apparent for the antioxidant capacity whereby the leaf extract of *J. curcas*, while the stem bark extract of *J. gossypiifolia*, both obtained by homogenizer assisted extraction showed the most significant free radical scavenging and reducing activity, and were also found to contain higher TPC and TFC. Furthermore, the homogenizer assisted extraction could be considered as a better extraction method than maceration to extract antioxidant compounds. The extracts were also tested in hypothalamic HypoE22 cells, and the pattern of gene expression coupled to bioinformatics analysis indicated anti-inflammatory and neuromodulatory effects, thus supporting further investigations, especially in experimental models of obesity. The data retained from the present study suggest the use of these two species as therapeutically important plants. Nevertheless, more intense investigations in vivo and under clinical settings could help to assess their respective safety profile.

## Figures and Tables

**Table 1:** Total bioactive contents (TPC and TFC) and total antioxidant capacity (phosphomolybdenum assay) of the tested extracts.

Species	Parts	Methods	TPC (mg GAE/g)	TFC (mg RE/g)
*J. curcas*	Leaves	HAE	48.95 ± 0.90 ^a^	13.99 ± 1.18 ^a^
		MAC	38.70 ± 0.53 ^b^	12.03 ± 0.21 ^b^
	Stem bark	HAE	6.72 ± 0.07 ^c^	2.67 ± 0.09 ^c^
		MAC	5.79 ± 0.06 ^c^	1.64 ± 0.01 ^c^
*J. gossypifolia*	Leaves	HAE	48.43 ± 0.31 ^b^	6.97 ± 0.32 ^d^
		MAC	42.62 ± 0.08 ^c^	11.04 ± 0.59 ^c^
	Stem bark	HAE	62.83 ± 2.05 ^a^	17.63 ± 0.34 ^a^
		MAC	49.05 ± 0.40 ^b^	12.71 ± 0.10 ^b^

Values are reported as mean ± SD. HAE: homogenizer-assisted extraction; MAC: maceration; TPC: Total phenolic content; TFC: Total flavonoid content; GAE: Gallic acid equivalent; RE: Rutin equivalent. Different letters in the same column indicate significant differences in the tested ex-tracts of each species (*p* < 0.05).

**Table 2:** Chemical composition of *J. curcas* leaves (HAE).

No.	Name	Formula	Rt	[M + H]^+^	[M − H]^−^	Literature
1 ^1^	Catechin	C15H14O6	14.17		289.07121	
2	Kynurenic acid	C10H7NO3	14.23	190.05042		
3	Bergenin	C14H16O9	14.52		327.07161	
4	Scopoletin-7-O-hexoside	C16H18O9	15.02	355.10291		
5 ^1^	Epiatechin	C15H14O6	17.61		289.07121	
6	Fraxetin (7,8-Dihydroxy-6-methoxycoumarin)	C10H8O5	17.68	209.04500		
7	Tomenin or isomer	C17H20O10	18.38	385.11348		
8 ^1^	Scopoletin (7-Hydroxy-6-methoxycoumarin)	C10H8O4	19.16	193.05009		
9	Hemiphloin (Naringenin-6-C-glucoside)	C21H22O10	19.84	435.12913		
10	Luteolin-C-hexoside-C-pentoside isomer 1	C26H28O15	19.87		579.13500	
11 ^1^	Taxifolin (Dihydroquercetin)	C15H12O7	19.92		303.05048	
12	Luteolin-C-hexoside-C-pentoside isomer 2	C26H28O15	20.03		579.13500	
13	Loliolide	C11H16O3	20.12	197.11777		
14	Apigenin-C-hexoside-O-hexoside	C27H30O15	20.29	595.16630		
15	Isohemiphloin (Naringenin-8-C-glucoside)	C21H22O10	20.39	435.12913		
16 ^1^	Coumarin	C9H6O2	20.55	147.04461		
17	Naringenin-C-hexoside isomer 3	C21H22O10	20.71	435.12913		
18	N-(2-Phenylethyl)acetamide	C10H13NO	20.76	164.10754		
19	Vicenin-1 (Apigenin-8-C-glucoside-6-C-xyloside)	C26H28O14	20.77	565.15574		
20	Orientin (Luteolin-8-C-glucoside)	C21H20O11	20.90	449.10839		[24]
21	Vicenin-3 (Apigenin-6-C-glucoside-8-C-xyloside)	C26H28O14	21.15	565.15574		
22	Isoorientin (Luteolin-6-C-glucoside)	C21H20O11	21.25	449.10839		
23 ^1^	Vitexin (Apigenin-8-C-glucoside)	C21H20O10	21.86	433.11347		[24]
24	Tomentin (6,7-Dimethoxy-5-hydroxycoumarin) or isomer	C11H10O5	22.22	223.06065		
25	Isovitexin (Apigenin-6-C-glucoside)	C21H20O10	22.77	433.11347		
26	Scoparin (Chrysoeriol-8-C-glucoside) or Isoscoparin (Chrysoeriol-6-C-glucoside)	C22H22O11	23.18	463.12404		
27 ^1^	Isoquercitrin (Quercetin-3-O-glucoside)	C21H20O12	23.44		463.08765	
28	Apigenin-C-pentoside isomer 1	C20H18O9	24.24	403.10291		
29 ^1^	Cosmosiin (Apigenin-7-O-glucoside)	C21H20O10	24.51	433.11347		
30	Apigenin-C-pentoside isomer 2	C20H18O9	24.82	403.10291		
31	Rhoifolin (Apigenin-7-O-neohesperidoside)	C27H30O14	24.93		577.15574	[24]
32	N-trans-Feruloyltyramine	C18H19NO4	25.15	314.13924		
33 ^1^	Eriodictyol (3′,4′,5,7-Tetrahydroxyflavanone)	C15H12O6	25.40		287.05556	
34	Dihydroactinidiolide	C11H16O2	27.08	181.12286		
35	Dihydroxy-dimethoxy(iso)flavone-C-hexoside	C23H24O11	27.31	477.13969		
36 ^1^	Quercetin (3,3′,4′,5,7-Pentahydroxyflavone)	C15H10O7	27.55		301.03483	
37 ^1^	Naringenin (4′,5,7-Trihydroxyflavanone)	C15H12O5	27.73		271.06065	
38	Jasmonic acid	C12H18O3	28.19		209.11777	
39	Jatrophenol I or II or II	C43H40O20	28.28		875.20347	[24]
40 ^1^	Luteolin (3′,4′,5,7-Tetrahydroxyflavone)	C15H10O6	28.43		285.03991	
41	Sebacic acid (Decanedioic acid)	C10H18O4	28.44		201.11268	
42	Quercetin-3-O-methyl ether	C16H12O7	28.78		315.05048	
43	Apigenin-C-pentoside isomer 3	C20H18O9	29.40	403.10291		
44 ^1^	Apigenin (4′,5,7-Trihydroxyflavone)	C15H10O5	30.26		269.04500	[24]
45	Jatrophenol I or II or II	C43H40O20	30.28		875.20347	[24]
46	Chrysoeriol (3′-Methoxy-4′,5,7-trihydroxyflavone)	C16H12O6	30.47		299.05556	
47	Undecanedioic acid	C11H20O4	31.32		215.12834	
48	3,3′,4,4′-Tetra-O-methylellagic acid	C18H14O8	32.63	359.07670		
49	Hydroxydodecenoic acid	C12H22O3	32.75		213.14907	
50	Dimethoxy-trihydroxy(iso)flavone	C17H14O7	33.30		329.06613	
51	Dodecanedioic acid	C12H22O4	33.74		229.14399	
52	Curcusone C or Curcusone D	C20H24O3	35.45	313.18037		[25]
53	Curcusone C or Curcusone D	C20H24O3	35.92	313.18037		[25]
54	12-Oxo phytodienoic acid or 13-Epi-12-oxo phytodienoic acid	C18H28O3	38.18		291.19603	
55	12-Oxo phytodienoic acid or 13-Epi-12-oxo phytodienoic acid	C18H28O3	39.80		291.19603	
56	Stearidonic acid	C18H28O2	40.12		275.20111	
57	Hydroxyoctadecatrienoic acid	C18H30O3	40.22		293.21167	
58	Hydroxyoctadecadienoic acid	C18H32O3	41.33		295.22732	
59	Stearidonic acid methyl ester	C19H30O2	42.09	291.23241		
60	Hydroxyhexadecenoic acid	C16H30O3	43.45		269.21167	
61 ^1^	α-Linolenic acid	C18H30O2	45.05		277.21676	[26]
62	Myristic acid	C14H28O2	45.16		227.20111	[26]
63	2-Hydroxyhexadecanoic acid	C16H32O3	45.22		271.22732	
64 ^1^	Linoleic acid	C18H32O2	46.05		279.23241	[26]
65	Palmitoleic acid	C16H30O2	46.30		253.21676	[26]
66	Palmitic acid	C16H32O2	46.98		255.23241	[26]
67 ^1^	Oleic acid	C18H34O2	47.10		281.24806	[26]
68	Stearic acid	C18H36O2	48.40		283.26371	[26]

^1^ Confirmed by standard.

**Table 3:** Chemical composition of *J. curcas* stem bark (HAE).

No.	Name	Formula	Rt	[M + H]^+^	[M −H]^−^	Literature
1	Scandoside methyl ester or isomer	C17H24O11	15.04		449.1295	
2	5-O-Feruloylquinic acid	C17H20O9	18.55		367.10291	
3	Loliolide	C11H16O3	20.09	197.11777		
4	Orientin (Luteolin-8-C-glucoside)	C21H20O11	20.88	449.10839		[24]
5	Isoorientin (Luteolin-6-C-glucoside)	C21H20O11	21.22	449.10839		
6 ^1^	Vitexin (Apigenin-8-C-glucoside)	C21H20O10	21.88	433.11347		[24]
7	Isovitexin (Apigenin-6-C-glucoside)	C21H20O10	22.80	433.11347		
8	Luteolin-7-O-glucoside (Cynaroside)	C21H20O11	22.89		447.09274	
9	Quercetin-O-rhamnosylpentoside	C26H28O15	23.30		579.13500	
10 ^1^	Isoquercitrin (Quercetin-3-O-glucoside)	C21H20O12	23.48		463.08765	
11 ^1^	Rutin (Quercetin-3-O-rutinoside)	C27H30O16	23.58	611.16122		
12	Tomatidine or isomer	C27H45NO2	24.45	416.35286		
13	Di-O-caffeoylquinic acid	C25H24O12	24.63		515.11896	
14 ^1^	Quercitrin (Quercetin-3-O-rhamnoside)	C21H20O11	25.03		447.09274	
15	Kaempferol-O-rhamnosylpentoside	C26H28O14	25.07		563.14009	
16	Dihydroactinidiolide	C11H16O2	27.09	181.12286		
17 ^1^	Quercetin (3,3′,4′,5,7-Pentahydroxyflavone)	C15H10O7	27.57		301.03483	
18	Jasmonic acid	C12H18O3	28.21		209.11777	
19	Sebacic acid (Decanedioic acid)	C10H18O4	28.44		201.11268	
20 ^1^	Luteolin (3′,4′,5,7-Tetrahydroxyflavone)	C15H10O6	28.45		285.03991	
21	Quercetin-3-O-methyl ether	C16H12O7	28.80		315.05048	
22	Solasodine or isomer	C27H43NO2	29.16	414.33721		
23 ^1^	Apigenin (4′,5,7-Trihydroxyflavone)	C15H10O5	30.29		269.04500	[24]
24	Undecanedioic acid	C11H20O4	31.33		215.12834	
25	Hydroxydodecenoic acid	C12H22O3	32.76		213.14907	
26	Dimethoxy-trihydroxy(iso)flavone	C17H14O7	33.33		329.06613	
27	Dodecanedioic acid	C12H22O4	33.76		229.14399	
28	Unidentified saponin 1	C42H66O15	34.36		809.43235	
29	Trihydroxyoctadecenoic acid	C18H34O5	35.46		329.23280	
30	Unidentified saponin 2	C42H66O15	35.73		809.43235	
31	Cynarasaponin C or isomer	C42H66O14	37.64		793.43744	
32	12-Oxo phytodienoic acid or 13-Epi-12-oxo phytodienoic acid	C18H28O3	38.20		291.19603	
33	12-Oxo phytodienoic acid or 13-Epi-12-oxo phytodienoic acid	C18H28O3	39.79		291.19603	
34	Stearidonic acid	C18H28O2	40.11		275.20111	
35	Hydroxyoctadecatrienoic acid	C18H30O3	40.20		293.21167	
36	Hexadecanedioic acid	C16H30O4	40.73		285.20659	
37	Hydroxyoctadecadienoic acid	C18H32O3	41.37		295.22732	
38 ^1^	α-Linolenic acid	C18H30O2	45.07		277.21676	[26]
39 ^1^	Linoleic acid	C18H32O2	46.06		279.23241	[26]
40	Palmitic acid	C16H32O2	46.99		255.23241	[26]
41 ^1^	Oleic acid	C18H34O2	47.11		281.24806	[26]
42	Stearic acid	C18H36O2	48.41		283.26371	[26]
43	Taraxasterol or isomer	C30H50O	50.86	427.39399		[27]
44	Taraxasterol or isomer	C30H50O	53.00	427.39399		[27]

^1^ Confirmed by standard.

**Table 4:** Chemical composition of *J. gossypifolia* leaves (HAE).

No.	Name	Formula	Rt	[M + H]^+^	[M − H]^−^	Literature
1	Quinic acid	C7H12O6	1.95		191.05557	
2 ^1^	Catechin	C15H14O6	14.20		289.07121	[26]
3	Kynurenic acid	C10H7NO3	14.24	190.05042		
4	Bergenin	C14H16O9	14.56		327.07161	
5	Biflorin	C16H18O9	15.08	355.10291		
6	Isobiflorin	C16H18O9	15.86	355.10291		
7 ^1^	Epiatechin	C15H14O6	17.63		289.07121	
8 ^1^	4-Coumaric acid	C9H8O3	18.63		163.03952	[26]
9	Isololiolide	C11H16O3	18.78	197.11777		
10 ^1^	Scopoletin (7-Hydroxy-6-methoxycoumarin)	C10H8O4	19.13	193.05009		
11	Isoschaftoside (Apigenin-6-C-arabinoside-8-C-glucoside)	C26H28O14	19.44	565.15574		[24]
12	Schaftoside (Apigenin-8-C-arabinoside-6-C-glucoside)	C26H28O14	19.78	565.15574		[24]
13	Luteolin-C-hexoside-C-pentoside isomer 1	C26H28O15	19.89		579.13500	
14 ^1^	Taxifolin (Dihydroquercetin)	C15H12O7	19.94		303.05048	
15 ^1^	Ferulic acid	C10H10O4	19.98		193.05009	[26]
16	Luteolin-C-hexoside-C-pentoside isomer 2	C26H28O15	20.05		579.13500	
17	Loliolide	C11H16O3	20.07	197.11777		
18	Vicenin-1 (Apigenin-8-C-glucoside-6-C-xyloside)	C26H28O14	20.73	565.15574		
19	Orientin (Luteolin-8-C-glucoside)	C21H20O11	20.83	449.10839		[24]
20	Vicenin-3 (Apigenin-6-C-glucoside-8-C-xyloside)	C26H28O14	21.10	565.15574		
21	Isoorientin (Luteolin-6-C-glucoside)	C21H20O11	21.17	449.10839		[24]
22 ^1^	Vitexin (Apigenin-8-C-glucoside)	C21H20O10	21.83	433.11347		[24]
23	Dihydrokaempferol (3,4′,5,7-Tetrahydroxyflavanone)	C15H12O6	22.51		287.05557	
24	Luteolin-C-pentoside	C20H18O10	22.56	419.09783		
25	Isovitexin (Apigenin-6-C-glucoside)	C21H20O10	22.75	433.11347		[24]
26	Luteolin-7-O-glucoside (Cynaroside)	C21H20O11	22.91		447.09274	
27	Scoparin (Chrysoeriol-8-C-glucoside) or Isoscoparin (Chrysoeriol-6-C-glucoside)	C22H22O11	23.20	463.12404		
28 ^1^	Isoquercitrin (Quercetin-3-O-glucoside)	C21H20O12	23.47		463.08765	
29	Apigenin-C-rhamnoside isomer 1	C21H20O9	23.62	417.11856		
30	Apigenin-C-pentoside isomer 1	C20H18O9	24.24	403.10291		
31	Apigenin-C-pentoside isomer 2	C20H18O9	24.91	403.10291		
32	Rhoifolin (Apigenin-7-O-neohesperidoside)	C27H30O14	24.95		577.15574	
33 ^1^	Eriodictyol (3′,4′,5,7-Tetrahydroxyflavanone)	C15H12O6	25.42		287.05556	
34	Apigenin-C-rhamnoside isomer 2	C21H20O9	26.19	417.11856		
35	Dihydroactinidiolide	C11H16O2	27.08	181.12286		
36	Dihydroxy-dimethoxy(iso)flavone-C-hexoside	C23H24O11	27.31	477.13969		
37 ^1^	Quercetin (3,3′,4′,5,7-Pentahydroxyflavone)	C15H10O7	27.57		301.03483	[26]
38 ^1^	Naringenin (4′,5,7-Trihydroxyflavanone)	C15H12O5	27.75		271.06065	
39	Jasmonic acid	C12H18O3	28.20		209.11777	
40	Jatrophenol I or II or II	C43H40O20	28.31		875.20347	
41 ^1^	Luteolin (3′,4′,5,7-Tetrahydroxyflavone)	C15H10O6	28.44		285.03991	[26]
42	Sebacic acid (Decanedioic acid)	C10H18O4	28.45		201.11268	
43	Quercetin-3-O-methyl ether	C16H12O7	28.80		315.05048	
44	Dimethoxy-tetrahydroxy(iso)flavone	C17H14O8	29.05		345.06105	
45 ^1^	Kaempferol (3,4′,5,7-Tetrahydroxyflavone)	C15H10O6	29.92		285.03991	[26]
46 ^1^	Apigenin (4′,5,7-Trihydroxyflavone)	C15H10O5	30.27		269.04500	[24]
47	Jatrophenol I or II or II	C43H40O20	30.32		875.20347	
48 ^1^	Isorhamnetin (3′-Methoxy-3,4′,5,7-tetrahydroxyflavone)	C16H12O7	30.42		315.05048	
49	Chrysoeriol (3′-Methoxy-4′,5,7-trihydroxyflavone)	C16H12O6	30.52		299.05556	
50	Methoxy-tetrahydroxy(iso)flavone	C16H12O6	30.93		299.05556	
51	Trihydroxy-trimethoxy(iso)flavone isomer 1	C18H16O8	31.09		359.07670	
52	Dimethoxy-trihydroxy(iso)flavone	C17H14O7	31.15		329.06613	
53	Undecanedioic acid	C11H20O4	31.32		215.12834	
54	Trihydroxy-trimethoxy(iso)flavone isomer 2	C18H16O8	31.74		359.07670	
55	Sakuranetin (4′,5-Dihydroxy-7-methoxyflavanone)	C16H14O5	32.54	287.09195		
56	Hydroxydodecenoic acid	C12H22O3	32.77		213.14907	
57	Trihydroxy-trimethoxy(iso)flavone isomer 3	C18H16O8	33.15		359.07670	
58	Trihydroxy-trimethoxy(iso)flavone isomer 4	C18H16O8	33.56		359.07670	
59	Dodecanedioic acid	C12H22O4	33.75		229.14399	
60	Dihydroxy-tetramethoxy(iso)flavone isomer 1	C19H18O8	33.85	375.10799		
61	Dihydroxy-tetramethoxy(iso)flavone isomer 2	C19H18O8	35.45	375.10799		
62	Hydroxy-tetramethoxy(iso)flavone	C19H18O7	37.04	359.11308		
63	Pinostrobin (5-Hydroxy-7-methoxyflavanone)	C16H14O4	37.08	271.09704		
64	Tetradecanedioic acid	C14H26O4	37.67		257.17529	
65	12-Oxo phytodienoic acid or 13-Epi-12-oxo phytodienoic acid	C18H28O3	38.21		291.19603	
66	12-Oxo phytodienoic acid or 13-Epi-12-oxo phytodienoic acid	C18H28O3	39.81		291.19603	
67	Stearidonic acid	C18H28O2	40.13		275.20111	
68	Hydroxyoctadecatrienoic acid	C18H30O3	40.22		293.21167	
69	Hydroxyoctadecadienoic acid	C18H32O3	41.36		295.22732	
70	Stearidonic acid methyl ester	C19H30O2	42.11	291.23241		
71	Hydroxyhexadecenoic acid	C16H30O3	43.46		269.21167	
72 ^1^	α-Linolenic acid	C18H30O2	45.06		277.21676	
73	2-Hydroxyhexadecanoic acid	C16H32O3	45.21		271.22732	
74 ^1^	Linoleic acid	C18H32O2	46.06		279.23241	
75	Palmitoleic acid	C16H30O2	46.28		253.21676	
76	Palmitic acid	C16H32O2	46.99		255.23241	
77 ^1^	Oleic acid	C18H34O2	47.09		281.24806	
78	Stearic acid	C18H36O2	48.38		283.26371	

^1^ Confirmed by standard.

**Table 5:** Antioxidant properties of the tested extracts.

Species	Parts	Methods	DPPH	ABTS	CUPRAC	FRAP	MCA	PDB
			(mg TE/g)				(mg EDTAE/g)	mmol TE/g
*J. curcas*	Leaves	HAE	124.70 ± 0.43 ^a^	149.12 ± 7.38 ^a^	256.21 ± 2.10 ^a^	97.03 ± 1.05 ^a^	10.98 ± 1.38 ^a^	2.57 ± 0.14 ^b^
		MAC	76.65 ± 0.95 ^b^	107.81 ± 1.38 ^b^	193.38 ± 0.66 ^b^	70.39 ± 0.22 ^b^	10.64 ± 0.81 ^a^	2.27 ± 0.12 ^b^
	Stem bark	HAE	6.89 ± 0.81 ^c^	20.20 ± 1.18 ^c^	24.90 ± 0.07 ^c^	15.19 ± 0.47 ^c^	5.28 ± 0.46 ^b^	3.34 ± 0.35 ^a^
		MAC	7.00 ± 0.20 ^c^	21.03 ± 1.33 ^c^	21.07 ± 0.32 ^d^	14.02 ± 0.18 ^c^	3.21 ± 0.27 ^b^	3.55 ± 0.16 ^a^
*J. gossypifolia*	Leaves	HAE	123.88 ± 1.05 ^b^	160.00 ± 1.62 ^b^	265.79 ± 0.59 ^b^	109.45 ± 1.43 ^c^	17.51 ± 0.71 ^b^	2.44 ± 0.11 ^a^
		MAC	124.29 ± 4.28 ^b^	149.65 ± 1.22 ^c^	245.10 ± 1.44 ^c^	101.32 ± 0.83 ^d^	18.98 ± 0.08 ^a^	2.01 ± 0.17 ^b^
	Stem bark	HAE	193.93 ± 0.23 ^a^	255.39 ± 3.00 ^a^	333.30 ± 5.32 ^a^	168.93 ± 1.17 ^a^	15.91 ± 0.15 ^c^	2.12 ± 0.09 ^ab^
		MAC	48.14 ± 0.12 ^c^	86.88 ± 0.96 ^d^	243.59 ± 1.64 ^c^	124.18 ± 1.38 ^b^	13.67 ± 0.65 ^d^	1.76 ± 0.18 ^b^

Values are reported as mean ± SD. TE: Trolox equivalent; EDTAE: EDTA equivalent; MCA: metal chelating activity; MAC: maceration; HAE: homogenizer assisted extraction. Different letters in same column indicate significant differences in the tested extracts of each species (*p* < 0.05).

**Table 6:** Enzyme inhibitory effects of the tested extracts.

Species	Parts	Methods	AChE	BChE	Tyrosinase	Amylase	Glucosidase
			(mg GALAE/g)		(mg KAE/g)	(mmol ACAE/g)	
*J. curcas*	Leaves	HAE	2.36 ± 0.25 ^a^	1.59 ± 0.12 ^c^	56.30 ± 3.24 ^a^	0.62 ± 0.02 ^a^	0.65 ± 0.01 ^b^
		MAC	Na	2.06 ± 0.20 ^b^	48.46 ± 0.57 ^b^	0.62 ± 0.01 ^a^	0.63 ± 0.01 ^c^
	Stem bark	HAE	2.04 ± 0.02 ^a^	3.35 ± 0.16 ^a^	40.51 ± 4.38 ^c^	0.31 ± 0.01 ^b^	0.81 ± 0.01 ^a^
		MAC	2.08 ± 0.03 ^a^	3.68 ± 0.15 ^a^	38.14 ± 0.54 ^c^	0.28 ± 0.03 ^b^	0.81 ± 0.01 ^a^
*J. gossypifolia*	Leaves	HAE	1.46 ± 0.13 ^b^	0.65 ± 0.07 ^a^	53.42 ± 4.15 ^a^	0.58 ± 0.01 ^a^	0.79 ± 0.01 ^b^
		MAC	1.12 ± 0.18 ^c^	Na	50.43 ± 0.81 ^a^	0.55 ± 0.01 ^b^	0.79 ± 0.01 ^ab^
	Stem bark	HAE	1.92 ± 0.13 ^a^	0.50 ± 0.07 ^b^	55.09 ± 3.54 ^a^	0.49 ± 0.01 ^c^	Na
		MAC	2.06 ± 0.03 ^a^	0.72 ± 0.06 ^a^	57.59 ± 0.33 ^a^	0.43 ± 0.01 ^d^	0.81 ± 0.01 ^a^

Values are reported as mean ± SD. GALAE: Galantamine equivalent; KAE: Kojic acid equivalent; ACAE: Acarbose equivalent; Na: not active. Different letters in same column indicate significant differences in the tested extracts of each species (*p* < 0.05).

## Data Availability

The data presented in this study are available on request from the corresponding author.

## Supplementary Materials

The following are available online at https://www.mdpi.com/article/10.3390/antiox10050792/s1, Table S1: Chemical composition of *J. curcas* leaves (HAE), Table S2: Chemical composition of *J. curcas* stem bark (HAE), Table S3: Chemical composition of *J. gossypifolia* leaves (HAE).
