# Supplementary material for: Chemical Composition and Biological Properties of Two Jatropha Species: Different Parts and Different Extraction Methods
Source: Antioxidants (Basel). 2021 May 17;10(5):792. doi: 10.3390/antiox10050792 (PMC8156752; doi:10.3390/antiox10050792)
Supplement: Supplementary file 1 [file antioxidants-10-00792-s001.zip › antioxidants-1210635-supplementary.pdf]

antioxidants

Article

Chemical composition and biological properties of two *Jatropha* species: Different parts and different extraction methods

Gokhan Zengin[1], Mohamad Fawzi Mahomoodally[2], Kouadio Ibrahime Sinan[1], Gunes Ak[1], Ouattara Katinan Etienne[3], Jugreet B. Sharmeen[2], Luigi Brunetti[4], Sheila Leone[4], Simonetta Cristina Di Simone[4], Lucia Recinella[4], Annalisa Chiavaroli[4], Luigi Menghini[4], Giustino Orlando[4*], József Jekő[5], Zoltán Cziáky[5], Claudio Ferrante[4]

[1]Physiology and Biochemistry Research Laboratory, Department of Biology, Science Faculty, Selcuk University, Campus, 42130 Konya, Turkey. gokhanzengin@selcuk.edu.tr (G.Z), sinankouadio@gmail.com (K.I.S) and akguneselcuk@gmail.com

[2]Department of Health Sciences, Faculty of Medicine and Health Sciences, University of Mauritius, 230 Réduit, Mauritius. f.mahomoodally@uom.ac.mu (M.F.M) and sharmeenjugs@gmail.com (J.B.S)

[3]Laboratoire de Botanique, UFR Biosciences, Université Félix Houphouët-Boigny, 00225 Abidjan, Côte d'Ivoire. katinan.etienne@gmail.com (O.K.E)

[4]Department of Pharmacy, Medicinal Plant Unit (MPU), Botanic Garden "Giardino dei Semplici", "G. d'Annunzio" University of Chieti-Pescara, Via dei Vestini, 66100 Chieti, Italy; luigi.brunetti@unich.it (L.B.); sheila.leone@unich.it (S.L.); disimonesimonetta@gmail.com (S.C.D.S.); lucia.recinella@unich.it (L.R.); annalisa.chiavaroli@unich.it (A.C.); luigi.menghini@unich.it (L.M.); giustino.orlando@unich.it (G.O.); claudio.ferrante@unich.it (C.F.);

[5]Agricultural and Molecular Research and Service Institute, University of Nyíregyháza, 4400 Nyíregyháza, Hungary cziaky.zoltan@nye.hu (Z.C.) and jjozsi@gmail.com (J.J).

* Correspondence: gokhanzengin@selcuk.edu.tr (G.Z); giustino.orlando@unich.it (G.O.)

Table S1. Chemical composition of *J. curcas* leaves (HAE).

No.	Name	Formula	Rt	[M + H]$^+$	[M - H]$^-$	Fragment 1	Fragment 2	Fragment 3	Fragment 4	Fragment 5	Literature
1[1]	Catechin	C15H14O6	14.17		289.07121	245.0821	205.0504	203.0709	151.0389	109.0282	
2	Kynurenic acid	C10H7NO3	14.23	190.05042		162.0552	144.0451	116.0498	89.0393		
3	Bergenin	C14H16O9	14.52		327.07161	312.0496	249.0400	234.0166	207.0293	192.0059	
4	Scopoletin-7-O-hexoside	C16H18O9	15.02	355.10291		193.0499	178.0263	165.0555	137.0598	133.0289	
5[1]	Epiatechin	C15H14O6	17.61		289.07121	245.0819	205.0501	203.0707	151.0386	109.0282	
6	Fraxetin (7,8-Dihydroxy-6-methoxycoumarin)	C10H8O5	17.68	209.04500		194.0215	181.0499	163.0393	149.0963	135.0443	
7	Tomenin or isomer	C17H20O10	18.38	385.11348		223.0603	208.0370	190.0263	162.0313		
8[1]	Scopoletin (7-Hydroxy-6-methoxycoumarin)	C10H8O4	19.16	193.05009		178.0263	165.0546	149.0599	137.0602	133.0287	
9	Hemiphloin (Naringenin-6-C-glucoside)	C21H22O10	19.84	435.12913		399.1081	339.0866	285.0757	219.0292	195.0292	
10	Luteolin-C-hexoside-C-pentoside isomer 1	C26H28O15	19.87		579.13500	489.1039	459.0942	399.0723	369.0620	339.0513	
11[1]	Taxifolin (Dihydroquercetin)	C15H12O7	19.92		303.05048	285.0408	199.0392	177.0180	151.0389	125.0231	
12	Luteolin-C-hexoside-C-pentoside isomer 2	C26H28O15	20.03		579.13500	489.1031	459.0969	399.0726	369.0620	339.0521	
13	Loliolide	C11H16O3	20.12	197.11777		179.1068	161.0962	135.1171	133.1015	107.0860	
14	Apigenin-C-hexoside-O-hexoside	C27H30O15	20.29	595.16630		433.1132	415.1028	337.0708	313.0708	283.0602	
15	Isohemiphloin (Naringenin-8-C-glucoside)	C21H22O10	20.39	435.12913		399.1088	339.0864	285.0759	219.0290	195.0292	
16[1]	Coumarin	C9H6O2	20.55	147.04461		119.0688	105.0704	103.0548	91.0548		
17	Naringenin-C-hexoside isomer 3	C21H22O10	20.71	435.12913		417.1179	399.1062	315.0861	219.0285	195.0292	
18	N-(2-Phenylethyl)acetamide	C10H13NO	20.76	164.10754		122.0968	105.0704	103.0550	90.9483	79.0550	
19	Vicenin-1 (Apigenin-8-C-glucoside-6-C-xyloside)	C26H28O14	20.77	565.15574		547.1452	529.1349	511.1242	427.1029	295.0605	
20	Orientin (Luteolin-8-C-glucoside)	C21H20O11	20.90	449.10839		431.0978	413.0870	353.0656	329.0658	299.0553	[26]
21	Vicenin-3 (Apigenin-6-C-glucoside-8-C-xyloside)	C26H28O14	21.15	565.15574		547.1456	529.1346	511.1232	379.0816	295.0605	

22	Isoorientin (Luteolin-6-C-glucoside)	C21H20O11	21.25	449.10839		431.0978	413.0873	353.0660	329.0658	299.0554	
23[1]	Vitexin (Apigenin-8-C-glucoside)	C21H20O10	21.86	433.11347		415.1030	397.0924	379.0815	313.0709	283.0604	[26]
24	Tomentin (6,7-Dimethoxy-5-hydroxycoumarin) or isomer	C11H10O5	22.22	223.06065		208.0367	190.0263	179.0338	162.0312	135.0443	
25	Isovitexin (Apigenin-6-C-glucoside)	C21H20O10	22.77	433.11347		415.1025	397.0920	337.0708	313.0708	283.0602	
26	Scoparin (Chrysoeriol-8-C-glucoside) or Isoscoparin (Chrysoeriol-6-C-glucoside)	C22H22O11	23.18	463.12404		445.1133	427.1027	367.0815	343.0815	313.0708	
27[1]	Isoquercitrin (Quercetin-3-O-glucoside)	C21H20O12	23.44		463.08765	301.0359	300.0280	271.0253	255.0302	178.9975	
28	Apigenin-C-pentoside isomer 1	C20H18O9	24.24	403.10291		385.0920	367.0815	349.0712	313.0710	283.0602	
29[1]	Cosmosiin (Apigenin-7-O-glucoside)	C21H20O10	24.51	433.11347		271.0602	153.0179	145.0283	119.0491		
30	Apigenin-C-pentoside isomer 2	C20H18O9	24.82	403.10291		385.0934	367.0814	337.0718	313.0709	283.0603	
31	Rhoifolin (Apigenin-7-O-neohesperidoside)	C27H30O14	24.93		577.15574	413.0882	269.0460	268.0382	117.0334	107.0125	[26]
32	N-trans-Feruloyltyramine	C18H19NO4	25.15	314.13924		177.0548	149.0598	145.0286	121.0651	103.0547	
33[1]	Eriodictyol (3′,4′,5,7-Tetrahydroxyflavanone)	C15H12O6	25.40		287.05556	151.0027	135.0441	125.0231	107.0125	83.0123	
34	Dihydroactinidiolide	C11H16O2	27.08	181.12286		163.1119	145.1014	135.1172	121.1015	107.0861	
35	Dihydroxy-dimethoxy(iso)flavone-C-hexoside	C23H24O11	27.31	477.13969		445.1138	427.1029	409.0922	325.0708	295.0604	
36[1]	Quercetin (3,3',4',5,7-Pentahydroxyflavone)	C15H10O7	27.55		301.03483	273.0402	178.9976	151.0025	121.0278	107.0129	
37[1]	Naringenin (4',5,7-Trihydroxyflavanone)	C15H12O5	27.73		271.06065	177.0200	165.0181	151.0026	119.0489	107.0126	
38	Jasmonic acid	C12H18O3	28.19		209.11777	165.1275	109.0643	59.0123			
39	Jatrophenol I or II or II	C43H40O20	28.28		875.20347	443.0987	431.0981	323.0567	311.0562	295.0613	[26]
40[1]	Luteolin (3',4',5,7-Tetrahydroxyflavone)	C15H10O6	28.43		285.03991	217.0503	199.0394	175.0394	151.0026	133.0283	
41	Sebacic acid (Decanedioic acid)	C10H18O4	28.44		201.11268	183.1021	157.1222	139.1118	137.0963	111.0801	
42	Quercetin-3-O-methyl ether	C16H12O7	28.78		315.05048	300.0280	271.0253	255.0300	243.0301		
43	Apigenin-C-pentoside isomer 3	C20H18O9	29.40	403.10291		385.0919	367.0816	337.0709	313.0710	283.0602	
44[1]	Apigenin (4',5,7-Trihydroxyflavone)	C15H10O5	30.26		269.04500	227.0344	225.0554	151.0026	149.0234	117.0331	[26]
45	Jatrophenol I or II or II	C43H40O20	30.28		875.20347	713.1809	443.0987	431.0986	323.0568	311.0559	[26]

46	Chrysoeriol (3'-Methoxy-4',5,7-trihydroxyflavone)	C16H12O6	30.47		299.05556	284.0331	256.0378	227.0349			
47	Undecanedioic acid	C11H20O4	31.32		215.12834	197.1178	153.1274	125.0957			
48	3,3',4,4'-Tetra-O-methylellagic acid	C18H14O8	32.63	359.07670		344.0530	343.0465	329.0294	313.0346	285.0396	
49	Hydroxydodecenoic acid	C12H22O3	32.75		213.14907	195.1386	183.1384				
50	Dimethoxy-trihydroxy(iso)flavone	C17H14O7	33.30		329.06613	314.0438	299.0205	271.0252			
51	Dodecanedioic acid	C12H22O4	33.74		229.14399	211.1336	185.1538	167.1433			
52	Curcusone C or Curcusone D	C20H24O3	35.45	313.18037		295.1697	285.1852	277.1586	267.1745	253.1591	[27]
53	Curcusone C or Curcusone D	C20H24O3	35.92	313.18037		295.1696	285.1848	277.1592	267.1746	253.1592	[27]
54	12-Oxo phytodienoic acid or 13-Epi-12-oxo phytodienoic acid	C18H28O3	38.18		291.19603	273.1867	247.2068	165.1275	150.1040	148.0881	
55	12-Oxo phytodienoic acid or 13-Epi-12-oxo phytodienoic acid	C18H28O3	39.80		291.19603	273.1862	247.2076	165.1277	150.1042	148.0882	
56	Stearidonic acid	C18H28O2	40.12		275.20111	257.1932	231.2104	177.1646	59.0125		
57	Hydroxyoctadecatrienoic acid	C18H30O3	40.22		293.21167	275.2023	235.1705	223.1337	195.1387	59.0124	
58	Hydroxyoctadecadienoic acid	C18H32O3	41.33		295.22732	277.2178	195.1386	171.1018			
59	Stearidonic acid methyl ester	C19H30O2	42.09	291.23241		259.2064	241.1948	217.1955	135.1171	93.0705	
60	Hydroxyhexadecenoic acid	C16H30O3	43.45		269.21167	251.2018	223.2065				
61[1]	α-Linolenic acid	C18H30O2	45.05		277.21676	59.0125					[28]
62	Myristic acid	C14H28O2	45.16		227.20111						[28]
63	2-Hydroxyhexadecanoic acid	C16H32O3	45.22		271.22732	253.2171	225.2222	223.2064			
64[1]	Linoleic acid	C18H32O2	46.05		279.23241						[28]
65	Palmitoleic acid	C16H30O2	46.30		253.21676						[28]
66	Palmitic acid	C16H32O2	46.98		255.23241						[28]
67[1]	Oleic acid	C18H34O2	47.10		281.24806						[28]
68	Stearic acid	C18H36O2	48.40		283.26371						[28]

Table S2. Chemical composition of *J. curcas* stem bark (HAE).

No.	Name	Formula	Rt	[M + H]+	[M - H]-	Fragment 1	Fragment 2	Fragment 3	Fragment 4	Fragment 5	Literature
1	Scandoside methyl ester or isomer	C17H24O11	15.04		449.12951[2]	241.0718	223.0604	179.0553	143.0338	101.0231	
2	5-O-Feruloylquinic acid	C17H20O9	18.55		367.10291	193.0502	191.0556	173.0447	134.0363	93.0331	
3	Loliolide	C11H16O3	20.09	197.11777		179.1070	161.0963	135.1172	133.1015	107.0861	
4	Orientin (Luteolin-8-C-glucoside)	C21H20O11	20.88	449.10839		431.0980	413.0871	353.0664	329.0660	299.0554	[26]
5	Isoorientin (Luteolin-6-C-glucoside)	C21H20O11	21.22	449.10839		431.0981	413.0873	353.0660	329.0659	299.0554	
6[1]	Vitexin (Apigenin-8-C-glucoside)	C21H20O10	21.88	433.11347		415.1028	397.0922	379.0811	313.0708	283.0604	[26]
7	Isovitexin (Apigenin-6-C-glucoside)	C21H20O10	22.80	433.11347		415.1029	397.0925	337.0710	313.0710	283.0605	
8	Luteolin-7-O-glucoside (Cynaroside)	C21H20O11	22.89		447.09274	327.0516	285.0412	284.0333			
9	Quercetin-O-rhamnosylpentoside	C26H28O15	23.30		579.13500	301.0365	300.0280	271.0253	255.0301	178.9974	
10[1]	Isoquercitrin (Quercetin-3-O-glucoside)	C21H20O12	23.48		463.08765	301.0358	300.0280	271.0252	255.0299	178.9983	
11[1]	Rutin (Quercetin-3-O-rutinoside)	C27H30O16	23.58	611.16122		303.0501	129.0549	85.0290	71.0498		
12	Tomatidine or isomer	C27H45NO2	24.45	416.35286		398.3424	273.2217	255.2111	161.1327	126.1280	
13	Di-O-caffeoylquinic acid	C25H24O12	24.63		515.11896	353.0882	335.0785	191.0555	179.0342	173.0446	
14[1]	Quercitrin (Quercetin-3-O-rhamnoside)	C21H20O11	25.03		447.09274	301.0359	300.0281	271.0253	255.0302	151.0026	
15	Kaempferol-O-rhamnosylpentoside	C26H28O14	25.07		563.14009	285.0410	284.0331	255.0301	227.0347	151.0025	
16	Dihydroactinidiolide	C11H16O2	27.09	181.12286		163.1119	145.1016	135.1172	121.1015	107.0860	
17[1]	Quercetin (3,3',4',5,7-Pentahydroxyflavone)	C15H10O7	27.57		301.03483	273.0418	178.9979	151.0027	121.0281	107.0126	
18	Jasmonic acid	C12H18O3	28.21		209.11777	165.1276	109.0645	59.0124			
19	Sebacic acid (Decanedioic acid)	C10H18O4	28.44		201.11268	183.1021	157.1223	139.1118	137.0960	111.0804	
20[1]	Luteolin (3',4',5,7-Tetrahydroxyflavone)	C15H10O6	28.45		285.03991	217.0503	199.0390	175.0390	151.0025	133.0283	
21	Quercetin-3-O-methyl ether	C16H12O7	28.80		315.05048	300.0280	271.0252	255.0301	243.0302		
22	Solasodine or isomer	C27H43NO2	29.16	414.33721		396.3268	271.2066	253.1954	157.1013	126.1280	
23[1]	Apigenin (4',5,7-Trihydroxyflavone)	C15H10O5	30.29		269.04500	227.0340	225.0542	151.0030	149.0231	117.0333	[26]
24	Undecanedioic acid	C11H20O4	31.33		215.12834	197.1179	153.1275	125.0958			
25	Hydroxydodecenoic acid	C12H22O3	32.76		213.14907	195.1386	183.1385				

26	Dimethoxy-trihydroxy(iso)flavone	C17H14O7	33.33		329.06613	314.0438	299.0203	271.0255			
27	Dodecanedioic acid	C12H22O4	33.76		229.14399	211.1334	185.1538	167.1431			
28	Unidentified saponin 1	C42H66O15	34.36		809.43235	527.3411	485.3279	355.2655			
29	Trihydroxyoctadecenoic acid	C18H34O5	35.46		329.23280	311.2230	293.2132	275.2021	201.1129	171.1018	
30	Unidentified saponin 2	C42H66O15	35.73		809.43235	647.3827	471.3482	453.3384	113.0231		
31	Cynarasaponin C or isomer	C42H66O14	37.64		793.43744	631.3851	587.3920	569.3859	497.3664	455.3532	
32	12-Oxo phytodienoic acid or 13-Epi-12-oxo phytodienoic acid	C18H28O3	38.20		291.19603	273.1861	247.2069	165.1273			
33	12-Oxo phytodienoic acid or 13-Epi-12-oxo phytodienoic acid	C18H28O3	39.79		291.19603	273.1872	247.2064	165.1276			
34	Stearidonic acid	C18H28O2	40.11		275.20111	257.1935	231.2121	177.1646	59.0125		
35	Hydroxyoctadecatrienoic acid	C18H30O3	40.20		293.21167	275.2025	235.1703	223.1335	195.1387	59.0125	
36	Hexadecanedioic acid	C16H30O4	40.73		285.20659	267.1968	223.2066				
37	Hydroxyoctadecadienoic acid	C18H32O3	41.37		295.22732	277.2178	195.1388	171.1017			
38[1]	α-Linolenic acid	C18H30O2	45.07		277.21676	59.0122					[28]
39[1]	Linoleic acid	C18H32O2	46.06		279.23241						[28]
40	Palmitic acid	C16H32O2	46.99		255.23241						[28]
41[1]	Oleic acid	C18H34O2	47.11		281.24806						[28]
42	Stearic acid	C18H36O2	48.41		283.26371						[28]
43	Taraxasterol or isomer	C30H50O	50.86	427.39399		409.3841	369.3524	289.2495	229.1951	81.0706	[29]
44	Taraxasterol or isomer	C30H50O	53.00	427.39399		409.3834	299.2737	217.1965	205.1948	95.0862	[29]

Table S3. Chemical composition of *J. gossypifolia* leaves (HAE).

No.	Name	Formula	Rt	[M + H]+	[M - H]-	Fragment 1	Fragment 2	Fragment 3	Fragment 4	Fragment 5	Literature
1	Quinic acid	C7H12O6	1.95		191.05557	173.0446	171.0289	127.0389	111.0074	85.028	
2[1]	Catechin	C15H14O6	14.20		289.07121	245.0818	205.0501	203.0708	151.0390	109.0282	[28]
3	Kynurenic acid	C10H7NO3	14.24	190.05042		162.0552	144.0446	116.0502	89.0394		
4	Bergenin	C14H16O9	14.56		327.07161	312.0499	249.0409	234.0171	207.0297	192.0059	
5	Biflorin	C16H18O9	15.08	355.10291		337.0929	319.0815	259.0606	235.0605	205.0500	
6	Isobiflorin	C16H18O9	15.86	355.10291		337.0944	319.0806	259.0605	235.0605	205.0499	
7[1]	Epiatechin	C15H14O6	17.63		289.07121	245.0818	205.0496	203.0705	151.0391	109.0282	
8[1]	4-Coumaric acid	C9H8O3	18.63		163.03952	119.0489	93.0331				[28]
9	Isololiolide	C11H16O3	18.78	197.11777		179.1069	161.0962	135.1171	133.1015	107.0860	
10[1]	Scopoletin (7-Hydroxy-6-methoxycoumarin)	C10H8O4	19.13	193.05009		178.0263	165.0549	149.0600	137.0601	133.0288	
11	Isoschaftoside (Apigenin-6-C-arabinoside-8-C-glucoside)	C26H28O14	19.44	565.15574		547.1419	511.1246	427.1036	409.0935	295.0605	[26]
12	Schaftoside (Apigenin-8-C-arabinoside-6-C-glucoside)	C26H28O14	19.78	565.15574		547.1456	511.1252	409.0931	379.0815	295.0605	[26]
13	Luteolin-C-hexoside-C-pentoside isomer 1	C26H28O15	19.89		579.13500	489.1032	459.0946	399.0727	369.0622	339.0505	
14[1]	Taxifolin (Dihydroquercetin)	C15H12O7	19.94		303.05048	285.0410	199.0397	177.0187	151.0386	125.0231	
15[1]	Ferulic acid	C10H10O4	19.98		193.05009	178.0264	149.0597	137.0236	134.0362	121.0285	[28]
16	Luteolin-C-hexoside-C-pentoside isomer 2	C26H28O15	20.05		579.13500	489.1046	459.0950	399.0722	369.0619	339.0517	
17	Loliolide	C11H16O3	20.07	197.11777		179.1069	161.0963	135.1171	133.1015	107.0860	
18	Vicenin-1 (Apigenin-8-C-glucoside-6-C-xyloside)	C26H28O14	20.73	565.15574		547.1447	529.1350	511.1252	427.1034	295.0608	
19	Orientin (Luteolin-8-C-glucoside)	C21H20O11	20.83	449.10839		431.0983	413.0875	353.0661	329.0662	299.0557	[26]
20	Vicenin-3 (Apigenin-6-C-glucoside-8-C-xyloside)	C26H28O14	21.10	565.15574		547.1465	529.1361	511.1252	379.0815	295.0605	
21	Isoorientin (Luteolin-6-C-glucoside)	C21H20O11	21.17	449.10839		431.0976	413.0881	353.0660	329.0659	299.0555	[26]
22[1]	Vitexin (Apigenin-8-C-glucoside)	C21H20O10	21.83	433.11347		415.1032	397.0926	379.0819	313.0710	283.0606	[26]
23	Dihydrokaempferol (3,4',5,7-Tetrahydroxyflavanone)	C15H12O6	22.51		287.05557	269.0456	259.0614	177.0549	151.0027	125.0231	
24	Luteolin-C-pentoside	C20H18O10	22.56	419.09783		401.0870	383.0767	365.0663	329.0660	299.0555	
25	Isovitexin (Apigenin-6-C-glucoside)	C21H20O10	22.75	433.11347		415.1038	397.0927	337.0711	313.0710	283.0606	[26]
26	Luteolin-7-O-glucoside (Cynaroside)	C21H20O11	22.91		447.09274	327.0513	285.0411	284.0332	256.0380	151.0027	

27	Scoparin (Chrysoeriol-8-C-glucoside) or Isoscoparin (Chrysoeriol-6-C-glucoside)	C22H22O11	23.20	463.12404		445.1133	427.1027	367.0818	343.0818	313.0710	
28[1]	Isoquercitrin (Quercetin-3-O-glucoside)	C21H20O12	23.47		463.08765	301.0360	300.0281	271.0254	255.0301	178.9978	
29	Apigenin-C-rhamnoside isomer 1	C21H20O9	23.62	417.11856		399.1084	381.0974	321.0759	297.0762	267.0655	
30	Apigenin-C-pentoside isomer 1	C20H18O9	24.24	403.10291		385.0928	367.0817	349.0707	313.0710	283.0606	
31	Apigenin-C-pentoside isomer 2	C20H18O9	24.91	403.10291		385.0928	367.0820	337.0714	313.0709	283.0606	
32	Rhoifolin (Apigenin-7-O-neohesperidoside)	C27H30O14	24.95		577.15574	413.0879	269.0459	268.0381	117.0333		
33[1]	Eriodictyol (3′,4′,5,7-Tetrahydroxyflavanone)	C15H12O6	25.42		287.05556	151.0026	135.0440	125.0231	107.0125	83.0123	
34	Apigenin-C-rhamnoside isomer 2	C21H20O9	26.19	417.11856		399.1076	381.0972	321.0762	297.0761	267.0654	
35	Dihydroactinidiolide	C11H16O2	27.08	181.12286		163.1120	145.1015	135.1172	121.1016	107.0861	
36	Dihydroxy-dimethoxy(iso)flavone-C-hexoside	C23H24O11	27.31	477.13969		445.1131	427.1029	409.0924	325.0709	295.0604	
37[1]	Quercetin (3,3',4',5,7-Pentahydroxyflavone)	C15H10O7	27.57		301.03483	273.0410	178.9978	151.0026	121.0282	107.0126	[28]
38[1]	Naringenin (4',5,7-Trihydroxyflavanone)	C15H12O5	27.75		271.06065	177.0184	165.0181	151.0027	119.0490	107.0126	
39	Jasmonic acid	C12H18O3	28.20		209.11777	165.1267	109.0643	59.0123			
40	Jatrophenol I or II or II	C43H40O20	28.31		875.20347	443.0990	431.0989	323.0567	311.0569	295.0616	
41[1]	Luteolin (3',4',5,7-Tetrahydroxyflavone)	C15H10O6	28.44		285.03991	217.0504	199.0396	175.0392	151.0026	133.0283	[28]
42	Sebacic acid (Decanedioic acid)	C10H18O4	28.45		201.11268	183.1021	157.1225	139.1117	137.0961	111.0803	
43	Quercetin-3-O-methyl ether	C16H12O7	28.80		315.05048	300.0281	271.0253	255.0301	243.0300	227.0344	
44	Dimethoxy-tetrahydroxy(iso)flavone	C17H14O8	29.05		345.06105	330.0388	315.0154	287.0204	271.0253	259.0251	
45[1]	Kaempferol (3,4',5,7-Tetrahydroxyflavone)	C15H10O6	29.92		285.03991	257.0458	229.0503	185.0603	151.0025	107.0124	[28]
46[1]	Apigenin (4',5,7-Trihydroxyflavone)	C15H10O5	30.27		269.04500	227.0351	225.0555	151.0027	149.0235	117.0333	[26]
47	Jatrophenol I or II or II	C43H40O20	30.32		875.20347	713.181	443.0991	431.0990	323.0566	311.0568	
48[1]	Isorhamnetin (3'-Methoxy-3,4',5,7-tetrahydroxyflavone)	C16H12O7	30.42		315.05048	300.0280	283.0258	271.0253	164.0103	151.0027	
49	Chrysoeriol (3'-Methoxy-4',5,7-trihydroxyflavone)	C16H12O6	30.52		299.05556	284.0331	256.0380	227.0340	151.0023	107.0128	
50	Methoxy-tetrahydroxy(iso)flavone	C16H12O6	30.93		299.05556	284.0332	256.0378	255.0301	227.0348		
51	Trihydroxy-trimethoxy(iso)flavone isomer 1	C18H16O8	31.09		359.07670	344.0542	329.0308	301.0358	286.0125	273.0410	
52	Dimethoxy-trihydroxy(iso)flavone	C17H14O7	31.15		329.06613	314.0439	299.0200	285.0411	271.0254	243.0299	
53	Undecanedioic acid	C11H20O4	31.32		215.12834	197.1179	153.1275	125.0956			
54	Trihydroxy-trimethoxy(iso)flavone isomer 2	C18H16O8	31.74		359.07670	344.0542	329.0308	314.0073	301.0360	286.0126	
55	Sakuranetin (4',5-Dihydroxy-7-methoxyflavanone)	C16H14O5	32.54	287.09195		269.0805	185.0444	167.0342	147.0443	119.0496	

56	Hydroxydodecenoic acid	C12H22O3	32.77		213.14907	195.1384	183.1384			
57	Trihydroxy-trimethoxy(iso)flavone isomer 3	C18H16O8	33.15		359.07670	344.0542	329.0309	314.0075	301.0360	286.0125
58	Trihydroxy-trimethoxy(iso)flavone isomer 4	C18H16O8	33.56		359.07670	344.0543	329.0309	315.0512	301.0359	286.0125
59	Dodecanedioic acid	C12H22O4	33.75		229.14399	211.1336	185.1541	167.1432		
60	Dihydroxy-tetramethoxy(iso)flavone isomer 1	C19H18O8	33.85	375.10799		360.0845	359.0772	345.0609	329.0657	313.0350
61	Dihydroxy-tetramethoxy(iso)flavone isomer 2	C19H18O8	35.45	375.10799		360.0844	359.0766	345.0610	327.0511	314.0426
62	Hydroxy-tetramethoxy(iso)flavone	C19H18O7	37.04	359.11308		344.0895	343.0816	329.0663	315.0866	301.0713
63	Pinostrobin (5-Hydroxy-7-methoxyflavanone)	C16H14O4	37.08	271.09704		173.0595	167.0342	165.0551	131.0495	103.0549
64	Tetradecanedioic acid	C14H26O4	37.67		257.17529	239.1652	195.1750	193.1005		
65	12-Oxo phytodienoic acid or 13-Epi-12-oxo phytodienoic acid	C18H28O3	38.21		291.19603	273.1873	247.2068	165.1276	150.1040	148.0882
66	12-Oxo phytodienoic acid or 13-Epi-12-oxo phytodienoic acid	C18H28O3	39.81		291.19603	273.1863	247.2063	165.1273	150.1042	148.0882
67	Stearidonic acid	C18H28O2	40.13		275.20111	257.1918	231.2115	177.1638	59.0123	
68	Hydroxyoctadecatrienoic acid	C18H30O3	40.22		293.21167	275.2023	235.1702	223.1341	195.1385	59.0125
69	Hydroxyoctadecadienoic acid	C18H32O3	41.36		295.22732	277.2178	195.1387	171.1018		
70	Stearidonic acid methyl ester	C19H30O2	42.11	291.23241		259.2069	241.1952	217.1946	135.1172	93.0705
71	Hydroxyhexadecenoic acid	C16H30O3	43.46		269.21167	251.2010	223.2065			
72[1]	α-Linolenic acid	C18H30O2	45.06		277.21676	59.0127				
73	2-Hydroxyhexadecanoic acid	C16H32O3	45.21		271.22732	253.2172	225.2222	223.2060		
74[1]	Linoleic acid	C18H32O2	46.06		279.23241					
75	Palmitoleic acid	C16H30O2	46.28		253.21676					
76	Palmitic acid	C16H32O2	46.99		255.23241					
77[1]	Oleic acid	C18H34O2	47.09		281.24806					
78	Stearic acid	C18H36O2	48.38		283.26371					
